# IoT–Cloud-Based Control of a Mechatronic Production Line Assisted by a Dual Cyber–Physical Robotic System Within Digital Twin, AI and Industry/Education 4.0/5.0 Frameworks

**DOI:** 10.3390/s26103194

**Published:** 2026-05-18

**Authors:** Adriana Filipescu, Georgian Simion, Adrian Filipescu, Dan Ionescu

**Affiliations:** 1 Department of Automation, “Dunarea de Jos” University of Galati, Domneasca Street, no. 47, 800008 Galati, Romania; adriana.filipescu@ugal.ro (A.F.); georgian.simion@ugal.ro (G.S.); dan.ionescu@ugal.ro (D.I.); 2 Doctoral School of Fundamental and Engineering Sciences, “Dunarea de Jos” University of Galati, Domneasca Street, no. 47, 800008 Galati, Romania

**Keywords:** A/D/R MPL, CPRS, Profinet, industrial communications, Ethernet, DT, AR/VR, IoT, cloud, PLC

## Abstract

**Highlights:**

**What are the main findings?**

- Feasibility of a DT-Driven A/D/R Production Line, effective integration of cyber–physical robotics systems and successful coordination of discrete and continuous processes.
- Enhanced human-in-the-loop interaction through AR/VR, AI, secure and scalable remote operation and industrial/educational relevance.

**What are the implications of the main findings?**

- Industrial transformation toward circular manufacturing and advancement of human–machine collaboration (Industry/Education 5.0).
- Pathway toward sustainable, resilient manufacturing systems, standardization and interoperability in cyber–physical systems.

**Abstract:**

This paper presents a Digital Twin (DT)-based framework for the control, monitoring, and intelligent optimization of an Assembly/Disassembly/Repair Mechatronic Production Line (A/D/R MPL), developed as a laboratory platform aligned with Industry/Education 4.0/5.0 paradigms. The A/D/R MPL is assisted by two complementary cyber–physical robotic systems: an Assembly/Disassembly/Replacement Cyber–Physical Robotic System (A/D/R CPRS), and a Mobile Cyber–Physical Robotic System (MCPRS), enabling both fixed and mobile intelligent operations. The CPRS is equipped with an industrial robotic manipulator (IRM) responsible for A/D/R tasks, while the A/D Mechatronic Line (A/D ML) consists of seven interconnected workstations (WS1–WS7) dedicated to storage, transport, quality control, and final product handling. MCPRS includes a wheeled mobile robot (WMR), carrying a robotic manipulator (RM) and Mobile Visual Servoing System (MVSS). Each workstation is connected to a local slave programmable logic controller (PLC), which communicates via PROFIBUS with a master PLC located at the CPRS level. Additional communication infrastructures include LAN PROFINET and LAN Ethernet for local integration, and WAN Ethernet connectivity enabled through open platform Communication-Unified Architecture (OPC-UA), ensuring interoperability, scalability, and remote accessibility. Also, MODBUS TCP as serial industrial communication is used between the master PLC and the MCPRS. Virtual environment supports task planning through Augmented Reality (AR) and real-time monitoring through Virtual Reality (VR). The system behaviour is modelled with synchronized hybrid Petri Nets (SHPNs) which describe the discrete and hybrid dynamics of A/D/R processes. Artificial intelligence (AI) techniques are integrated into the DT framework for optimal task scheduling and adaptive decision-making. As a laboratory-scale implementation, the proposed system provides a comprehensive platform for experimentation, validation, and education. It supports Education 4.0/5.0 objectives by facilitating hands-on learning, human–machine interaction, and the integration of emerging technologies such as AI, Digital Twins, AR/VR, and cyber–physical systems. At the same time, it embodies Industry 4.0/5.0 principles, including interoperability, decentralization, sustainability, robustness, and human-centric design.

## 1. Introduction

The increasing demand for flexible, sustainable, and intelligent manufacturing systems has driven the convergence of Digital Twins (DTs), cyber–physical robotic systems (CPRSs), mobile robotics, and cloud-based technologies within the paradigms of Industry 4.0 and the emerging Industry/Education 5.0. Assembly, disassembly, and repair processes play a critical role in enabling circular manufacturing by supporting component recovery, reuse, and remanufacturing. These requirements are especially relevant in complex industrial domains, such as automotive body, gearbox, and engine block manufacturing, where high levels of automation coexist with stringent quality and sustainability constraints.

In this context, this paper presents a Digital Twin-based control framework for an assembly, disassembly, and repair mechatronic production line (A/D/R MPL) assisted by a dual cyber–physical robotic system, CPRS and MCPRS. The proposed framework extends conventional fixed-robot production lines by integrating mobile manipulation, real-time data-driven intelligence, and immersive human–machine interaction, thereby increasing system autonomy, flexibility, and efficiency. Although the CPRS can also function as an autonomous robotic cell that can perform assembly, disassembly and replacement, it is still used as a workstation attached to the A/D ML, which can perform assembly, disassembly and repair operations with component recovery as a MPL assisted by the MCPRS [1,2,3,4,5,6,7,8,9].

### 1.1. Motivation and Background

Across industries, DT technology has emerged as a powerful tool for bridging the physical and virtual worlds of manufacturing systems. DT represents a synchronized digital replica of a physical asset, process, or system, continuously updated through real-time data acquired from sensors and control devices. When combined with cloud computing, AI, and Internet of Things (IoT) technologies, DT enables advanced capabilities such as predictive maintenance, adaptive control, remote supervision, and what-if analysis.

MCPRS further enhance manufacturing flexibility by enabling autonomous transportation, manipulation, and coordination across distributed workstations. MCPRSs intertwine hardware and software components, combining mobile platforms, robotic manipulators, sensing systems, and computational intelligence to operate adaptively in dynamic environments. Such systems are increasingly applied in manufacturing, intelligent service robotics, education, and IoT–Cloud-based control, particularly where local resources are insufficient to handle complex tasks.

Despite these advances, most industrial A/D/R production lines still rely on fixed robotic manipulators and rigid control architectures. This limits their ability to support dynamic reconfiguration, efficient recovery of components, and seamless human–robot collaboration. The work presented in this paper addresses these limitations by integrating DT-driven control, mobile manipulation, and AI-enabled decision-making into a unified A/D/R MPL [2,3,4,5,6,7,8,9,10,11,12,13].

### 1.2. Overview of the Proposed A/D/R MPL Assisted by Dual CPRS

**Figure 1:**
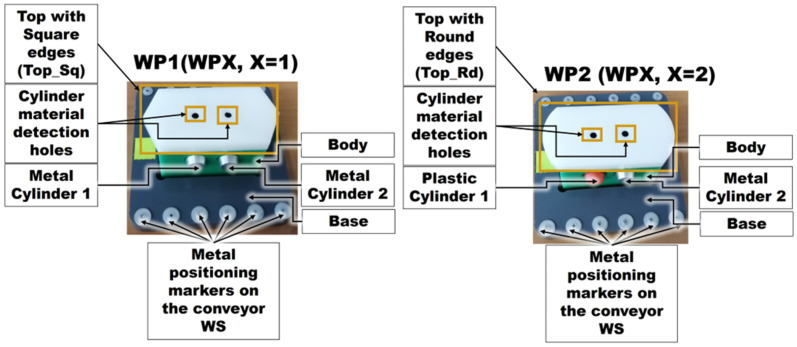
WP1-Top with Square edges (Top_Sq) and WP2-Top with Round edges (Top_Rd).

**Figure 2:**
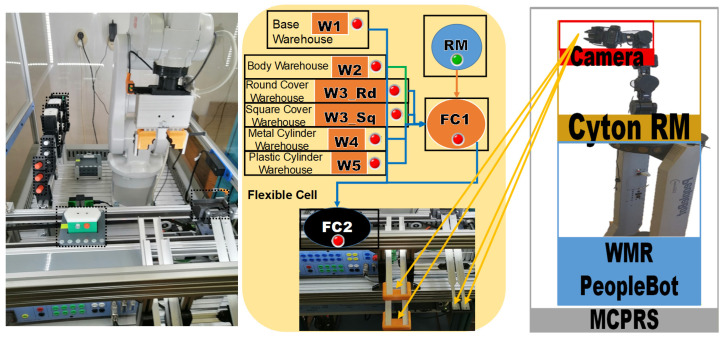
A/D/Replacement CPRS assisted by MCPRS.

**Figure 3:**
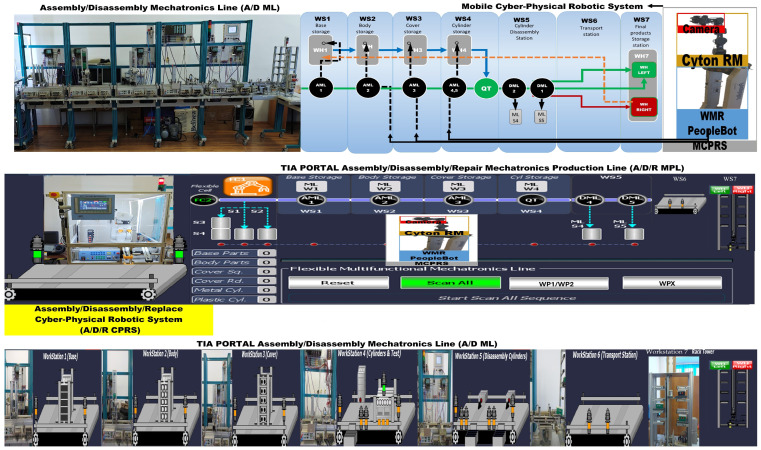
A/D ML and interaction with the MCPRS.

The proposed A/D/R MPL integrates an A/D/R CPRS) with a 7-WS A/D ML. The A/D/R CPRS is equipped with a six-degree-of-freedom (6-DOF) IRM, which performs flexible assembly, disassembly, and replacement operations. The A/D ML provides distributed processing, transportation, storage, and quality-testing functionalities across multiple workstations (Shown in Figure 1, Figure 2 and Figure 3).

The system is assisted by MCPRS composed of a 2DW/1FW WMR, a 7-DOF RM, and a MVSS mounted on the end-effector and equipped with a vision camera. The MCPRS autonomously transports workpieces (WPs) and recovered components between the A/D/R CPRS, the A/D ML, and warehouse areas, enabling dynamic material handling, component recovery, and circular manufacturing operations.

The proposed A/D/R MPL assembles two categories of workpieces. The first category corresponds to WP1/WP2 workpieces assembled directly inside the A/D/R CPRS. These workpieces are considered compliant regardless of the cylinder material because their configuration is predefined through the Human–Machine Interface (HMI). Consequently, WP1/WP2 workpieces are not subjected to the quality inspection stage and are directly transferred and stored in the WHL warehouse of WS7.

The second category corresponds to WPX workpieces assembled on the A/D ML. In this case, the cylinders used during assembly are randomly selected from the WH4 warehouse of WS4 and may be metallic or plastic. Because of this random material distribution, all WPX workpieces assembled on the A/D ML are subjected to a quality inspection process in WS7. Based on the detected cylinder material combination, the assembled WPX workpieces are classified into three categories:

- Compliant workpieces, when both cylinders are metallic,
- Fully compromised workpieces, when both cylinders are plastic,
- Repairable workpieces, when one cylinder is metallic and the other is plastic.

Compliant WPX workpieces are considered acceptable products and are stored in the WHL warehouse of WS7.

Fully compromised workpieces are considered unsuitable for use and are stored in the WHR warehouse of WS7 before entering the disassembly process in the A/D/R CPRS. During disassembly, the reusable components are recovered and transported by the MCPRS toward their corresponding warehouses in the A/D ML for future reuse in assembly operations. The only exception is the Base (Pallet), which is transported through the conveyor system toward warehouse WH1 in WS1 of the A/D ML.

Repairable workpieces are stored in the VHR warehouse of WS7 and are subsequently transferred to the A/D/R CPRS for replacement operations. In this case, the defective plastic cylinder is replaced with a metallic cylinder. The removed plastic cylinder is then recovered by the MCPRS and transported toward warehouse WH4 in WS4 of the A/D ML for storage and reuse management. Therefore, the proposed process currently focuses on replacement operations rather than complete maintenance procedures [9,10,11,12,13,14,15,16,17,18,19,20].

The trigger relationship among the assembly, disassembly, and replacement processes is governed by the quality inspection results obtained after WPX assembly. Thus, the process flow can be summarized as follows:

$$\begin{array}{l} \mathrm{WPX}\;\mathrm{Assembly}\to \mathrm{Quality}\;\mathrm{Inspection}\\ \left\{\begin{array}{cc} \mathrm{Both}\;\mathrm{metallic}\;\mathrm{cylinders} & \Rightarrow \mathrm{Compliant}\;\mathrm{WPX}\to WHL\\ \mathrm{Both}\;\mathrm{plastic}\;\mathrm{cylinders} & \Rightarrow \mathrm{Fully}\;\mathrm{compromised}\;\mathrm{WPX}\to \mathrm{Disassembly}\\ \mathrm{Mixed}\;\mathrm{cylinders} & \Rightarrow \mathrm{Repairable}\;\mathrm{WPX}\to \mathrm{Replacement} \end{array}\right. \end{array}$$

Consequently, the disassembly and replacement operations are not independent processes but are conditionally triggered according to the quality evaluation of the assembled WPX workpieces. This closed-loop material recovery mechanism enables systematic reuse of components, intelligent recycling of defective parts, and dynamic reconfiguration of the production flow, directly supporting circular manufacturing, sustainability, and Industry/Education 4.0/5.0 objectives.

### 1.3. IoT, AI, and Cloud-Based Integration

The ABB IRB integrated into the CPRS performs intricate A/D/R tasks using data acquired from IoT sensors monitoring component positioning, alignment, and applied forces during joining operations. These data are processed in real time to adapt robot behaviour to process variations using AI-driven machine learning (ML) models hosted at the edge and in the cloud. To enhance autonomy, adaptability, and robustness of the proposed DT–based A/D/R MPL assisted by a dual CPRS, advanced learning and control techniques can be integrated at different layers of the system. Reinforcement learning, fuzzy logic-based learning, and fractional-order learning provide complementary capabilities for handling uncertainty, nonlinearity, and time-varying dynamics inherent in assembly, disassembly, and repair processes.

The system incorporates a wide range of edge IoT devices, including PLCs, embedded computers, image processors, robotic controllers, communication modules, and servo drives. These devices are interconnected through local PROFINET and Ethernet networks and linked to the Internet via WAN connectivity. IoT sensors and cameras installed on the A/D/R MPL, CPRS, and MCPRS capture real-time operational data during assembly, disassembly, and repair tasks.

IoT gateways aggregate and securely transmit data to cloud services, enabling advanced analytics, visualization, predictive maintenance, and decision support. A virtual private network (VPN) ensures secure, encrypted communication between the manufacturing cell, cloud platform, and authorized remote users, protecting the system against cyber threats while enabling remote monitoring and control [6,8,10].

### 1.4. Digital Twin, AR/VR, and Petri Net-Based Control

The entire platform follows a DT paradigm aligned with Industry/Education 4.0/5.0 principles. The DT provides a real-time synchronized virtual representation of the physical A/D/R MPL, enabling continuous monitoring, simulation, and optimization.

Within the DT framework, Augmented Reality (AR) is used for task planning and human-in-the-loop interaction. Through AR-enhanced Human–Machine Interfaces (HMIs) and Node-RED-based visualization tools, users can observe real-time system states, interact with virtual workstations, and modify task sequences intuitively. Virtual Reality (VR) is employed for immersive simulation and validation based on SHPN models.

Synchronized timed Petri Nets (STPNs) are used to model and control discrete assembly operations at each workstation, while SHPNs with Continuous Petri Nets (CPNs) coordinate disassembly and repair. MCPRS assists transportation. CPNs govern the continuous motion of the MCPRS, representing the dynamic of the WMR, robotic manipulator, and MVSS. This unified modelling approach enables seamless coordination of discrete-event processes and continuous motion within a single formal framework [5,6,7,8,9,10,11,12,13,14,15,16,17,18,19,20,21,22,23,24,25,26,27,28,29].

### 1.5. Control Architecture, SCADA, and HMIs

A hierarchical control architecture is adopted, in which each WS is interfaced with a slave programmable logic controller (PLC) connected via PROFIBUS to a master PLC embedded within A/D/R CPRS. Additional devices are integrated through local PROFINET and Ethernet networks, while interoperability across all system layers is ensured via OPC- UA. This protocol is considered the standard for Industry 4.0-enabled facilities.

A supervisory control and data acquisition (SCADA) system provides real-time monitoring and control of the A/D/R MPL and MCPRS, collecting data from IoT sensors, actuators, and controllers. SCADA interfaces display key information such as workpiece positions, actuator states, and task progression, enabling informed decision-making.

Multiple HMIs are implemented for both local and remote interaction:

- HMI–Assembly, dedicated to monitoring and control of CPRS and A/D ML assembly operations,
- HMI–Disassembly, focused on disassembly processes and component recovery by the MCPRS,
- HMI–Repair, dedicated to repair operations and bad component replacement/recovery tasks.

These HMIs integrate data from SCADA, DT models, and IoT sensors, enhancing operational clarity, educational value, and human–machine collaboration [30,31,32,33,34,35,36,37,38,39,40,41,42,43,44,45,46,47].

### 1.6. Contributions and Scope

Although the proposed system is implemented and validated on a laboratory-scale platform, it has direct relevance to real-world industrial applications, particularly in automotive manufacturing and other sectors where component recovery and reuse are essential. By integrating MCPRSs into traditionally fixed robotic production lines, this work extends the degree of automation, flexibility, and sustainability.

The main contributions of this paper are:

- A DT-based control framework for an A/D/R MPL assisted by an MCPRS,
- Integration of IoT, AI, cloud, AR/VR, and Petri Net-based modelling within a unified architecture,
- Formal coordination of discrete and continuous processes using STPNs, SHPNs, and CPNs,
- Support for circular manufacturing and Education 5.0 through human-in-the-loop interaction and remote accessibility.

### 1.7. Structure of the Paper

- Section 1 (Introduction) presents the motivation, background, and overall vision of the A/D/R MPL assisted by dual CPRS, highlighting the integration of IoT–Cloud, AI, Digital Twin, AR/VR, and Petri Net-based control, together with the main contributions.
- Section 2 (Hardware and DT architecture) describes the physical system (A/D/R CPRS, A/D ML, MCPRS) and the distributed control infrastructure, including edge–cloud communication, SCADA/HMI integration, and the Digital Twin architecture.
- Section 3 (Virtual layer of DT) introduces the virtual layer based on AR/VR and SHPN modelling, detailing task planning, synchronization, and the unified modelling of assembly, disassembly, repair, and MCPRS operations.
- Section 4 (Real-time control and implementation) presents the real counterpart, including communication architecture, AI-based perception, MCPRS control by terminal twisting Sliding Mode Control (TTSMC), invers kinematic control (IKC), and image based visual servoing (IBSV)-based, $H_{\infty }$-robustness, synchronization via Petri Nets, and experimental real-time validation.
- Section 5 (AI optimization layer) presents the artificial intelligence optimization layer integrated into the IoT–Cloud-based architecture of the A/D/R MPL assisted by the dual Cyber–Physical Robotic System (CPRS and MCPRS). The proposed layer combines reinforcement learning (RL), type-3 fuzzy logic system (T3FLS), and Fractional-Order Control (FOC).
- Section 6 (Discussion) analyses system performance and broader implications, including circular manufacturing, sustainability, AI-based optimization, integration within Industry/Education 4.0/5.0 frameworks, cybersecurity protection.
- Section 7 (Conclusions) summarizes the main results, validates the proposed approach, and outlines contributions and future research directions.

## 2. Hardware Architecture of the A/D/R MPL and MCPRS

### 2.1. System Overview

The experimental platform is a multifunctional A/D/R MPL designed to support flexible manufacturing operations such as product assembly, disassembly of defective products, and repair through component replacement. The architecture integrates industrial robotics, distributed PLC control, artificial vision, edge computing, and cloud connectivity, forming a cyber–physical production system (CPPS).

The system is composed of three main subsystems:

- Assembly/Disassembly/Replacement Cyber–Physical Robotic System (A/D/R CPRS),
- Assembly/Disassembly Mechatronics Line (A/D ML),
- Mobile Cyber–Physical Robotic System (MCPRS).

These subsystems cooperate to perform the manufacturing and recovery operations associated with two workpiece types (WP1 and WP2), each of them assembled of five components, shown in Figure 1.

The CPRS performs assembly and repair operations using an industrial manipulator, while the A/D ML provides transport, quality testing, sorting, and storage functionalities. The MCPRS enables flexible manipulation and transport of reusable components within the system.

### 2.2. Assembly/Disassembly/Replacement Cyber–Physical Robotic System

The A/D/R CPRS represents a flexible robotic cell designed for performing assembly, disassembly, and component replacement operations. The core element of this subsystem is a 6-DOF industrial manipulator, which performs automated part handling and assembly operations. The CPRS includes the following main components as is shown in Figure 2:

- 6-DOF ABB IRB 120 (ABB Ltd., Zurich, Switzerland), industrial manipulator,
- ABB IRC5C robot controller,
- Electric gripper for component manipulation
- Siemens S7-1214C PLC,
- Component warehouses,
- Conveyor transport modules,
- Artificial vision subsystem.

Three 3D cameras monitor the warehouse areas to detect part availability and support automated manipulation. The artificial vision system consists of three IFM O3R222 cameras (ifm electronic gmbh, Essen, Germany) connected to an OVP800 video processing unit (VPU), (ifm electronic gmbh, Essen, Germany). These cameras perform part detection tasks for the Base (Pallet), Body, Top (Cover) implemented using ROS2 Humble (Humble Hawksbill (LTS Release)) and the IFM3D_ROS2 software library (1.4.1), enabling integration with higher-level control and monitoring services.

Figure 2 illustrates the left-side subsystem of the A/D/R MPL, namely the Assembly/Disassembly/Replace Cyber–Physical Robotic System (A/D/R CPRS). The CPRS consists of an industrial robotic manipulator operating inside a flexible robotic cell and interacting with multiple component warehouses. The warehouses include W1-Base (Pallet), W2-Body, W3_Rd-round Top (Cover), W3_Sq-square Top (Cover), W4-Metal Cylinder, and W5-Plastic Cylinders. The robotic manipulator retrieves components sequentially from the warehouses and performs assembly operations in two working areas: FC1, where the upper part of the workpiece (body, top, and cylinders) is assembled, and FC2, where the base component is placed on the conveyor. After assembling the upper structure in FC1, the robot places it onto the base located at FC2, completing the product assembly before transferring the workpiece to the mechatronic production line.

### 2.3. Assembly/Disassembly Mechatronics Line

The A/D ML is the modular Hera&Horstmann mechatronic production line consisting of of several interconnected workstations responsible for part feeding, assembly support, product transportation, quality inspection, and storage. The production line includes the following stations:

- WS1 with WH1-Pallet warehouse,
- WS2 with WH2-Body warehouse,
- WS3—with WH3-Top warehouse,
- WS4—with WH4-Cylinder warehouse and QT—quality testing station,
- WS5—bidirectional transport station with two locations for cylinder disassembly DML1 and DML2 (not used in this paper),
- WS6—multidirectional transport station,
- WS7—with WHL good WP storage and WHR for temporary storage of totally compromised or repairable items.

Products assembled in the system consist of modular components including a base, body, top element, and two cylinders, which may be either metal or plastic.

The WS4 station performs quality verification based on the configuration of the assembled cylinders. Products that satisfy the quality criteria are transported to the left storage rack (WHL) of WS7, while defective products are directed to the right storage rack (WHR). Defective products may subsequently undergo disassembly or repair operations within the CPRS.

Figure 3 presents the right-side subsystem of the A/D/R MPL, namely the A/DML, which consists of a sequence of automated workstations used for component supply, transportation, quality inspection, disassembly, and storage of workpieces. The line includes seven stations: WS1 (base storage), WS2 (body storage), WS3 (cover storage), WS4 (cylinder storage and quality testing), WS5 (cylinder disassembly station), WS6 (transport station), and WS7 (final product storage). Products move sequentially along the line through automated conveyor modules (AML and DML). At the quality test station (QT), products are evaluated based on the material configuration of the cylinders. Good products are transported to the left storage rack (WHL), while defective products are directed to the right storage rack (WHR). The figure also shows the MCPRS composed of PeopleBot WMR as mobile platform equipped with Cyton 1500 RM and an eye-in-hand Logitech camera (Logitech International S.A., Lausanne, Switzerland), which assists in manipulation and component recovery tasks.

### 2.4. Mobile Cyber–Physical Robotic System

**Figure 4:**
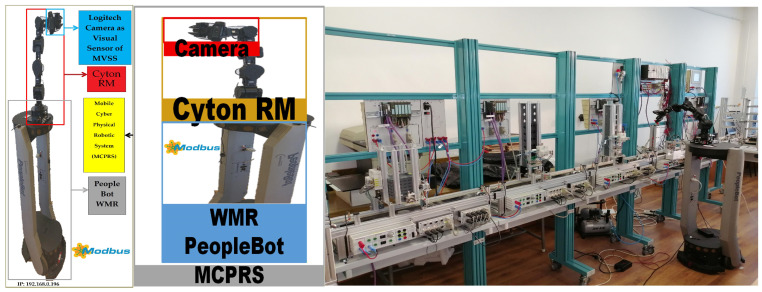
Architecture of MCPRS (PeopleBot WMR + Cyton RM + MVSS.

The MCPRS enhances the flexibility of the production system by providing mobile manipulation and component recovery capabilities. The platform, in Figure 4, consists of:

- PeopleBot wheeled mobile robot (WMR),
- 7-DOF Cyton 1500 (Robai Corporation, Cambridge, MA, USA) robotic manipulator,
- Eye-in-Hand Mobile Visual Servoing System (MVSS).

The mobile robot uses a two-drive-wheel/one-free-wheel (2DW/1FW) configuration and communicates with the production system through wireless networking. MCPRS performs tasks such as transport of reusable components, manipulation of recovered components, assistance in disassembly operations, and flexible interaction with production stations.

### 2.5. Edge Computing and Communication Infrastructure

**Figure 5:**
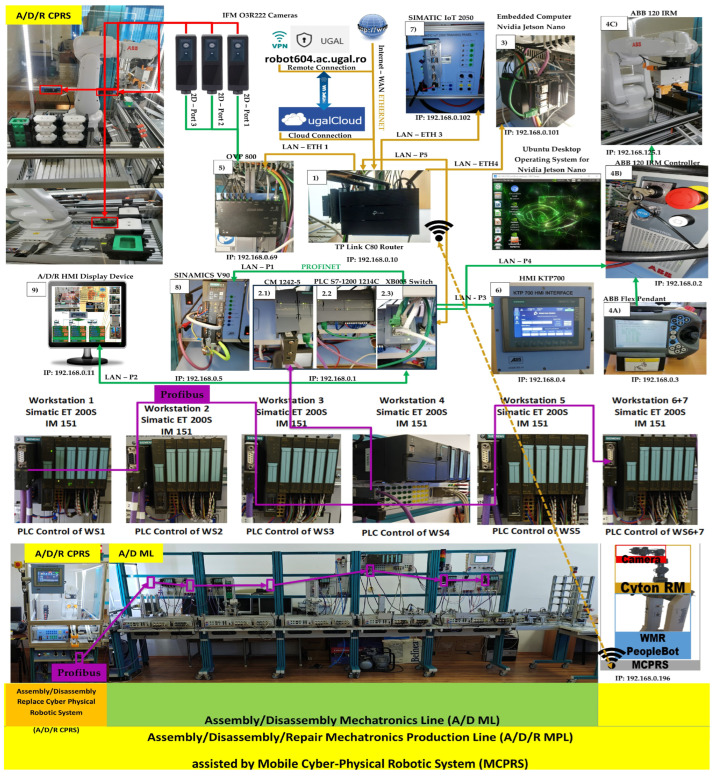
Devices and communication infrastructure.

To support cyber–physical integration and remote operation, the system incorporates several edge computing devices and industrial communication network. The edge layer, shown in Figure 5, includes:

- NVIDIA Jetson Nano embedded computer with UBUNTU 18.04 operation system,
- SIMATIC IoT 2050 industrial gateway is an edge and open-source device manufactured by Siemens (Alachua, FL, USA) that uses Linux-based operative system. Node-RED can run in the IoT2050.
- OVP800 video processing unit.
- TP-Link Archer Wi-Fi router.

The Jetson Nano executes Node-RED services, containerized applications, and remote visualization tools. These components enable integration between industrial devices, vision systems, and cloud-based monitoring platforms. Node-RED is an open-source middleware which is being applied for data handling and visualization.

Multiple communication networks are used within the system:

- PROFINET for real-time industrial communication,
- PROFIBUS-DP for communication between distributed PLCs,
- LAN Ethernet for communication between computing devices and SCADA systems,
- WAN Ethernet for remote access and cloud connectivity,
- MODBUS for serial communication between master PLC and MCPRS.

### 2.6. Distributed Control Architecture

**Figure 6:**
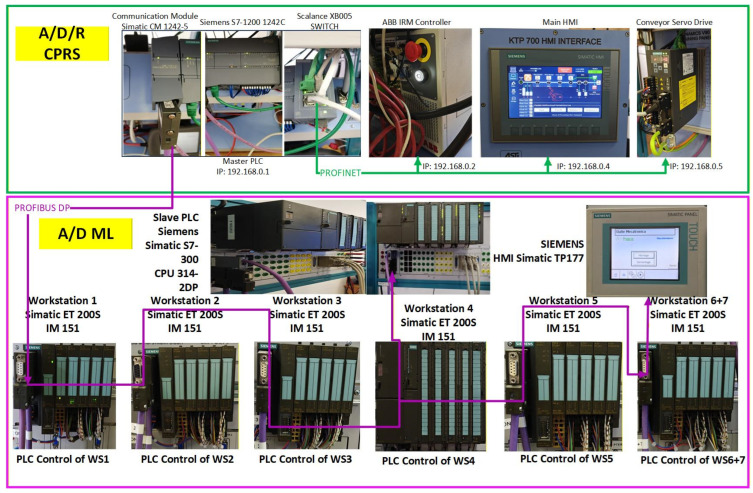
Distributed communication and control architecture.

The production system adopts a distributed control architecture. The main supervisory controller is a Siemens S7-1214C PLC, responsible for coordinating the operations of the robotic cell and the mechatronics line. Each workstation of the A/D ML is controlled by Siemens S7-300 PLCs equipped with ET200S distributed I/O modules. Communication between these controllers is achieved using PROFIBUS-DP, enabling synchronized control of transport, assembly, and inspection processes as is shown in Figure 6. The legacy equipment, PLC S7-300, ET200S and TP177, and the fieldbus Profibus, are integrated in this paper in an efficient manner, being a benefit of the developed architecture.

### 2.7. Multilevel Monitoring and Control Structure

The overall architecture follows a five-level hierarchical control structure:

- Remote operation level—cloud services, VPN access, and remote SCADA dashboards,
- Local operation level—local SCADA systems and operator HMIs,
- Communication level—IoT gateways, routers, and edge computing devices,
- Control level—PLCs, robot controllers, and industrial drives,
- Process level—physical manufacturing equipment including CPRS, A/D ML, and MCPRS.

**Figure 7:**
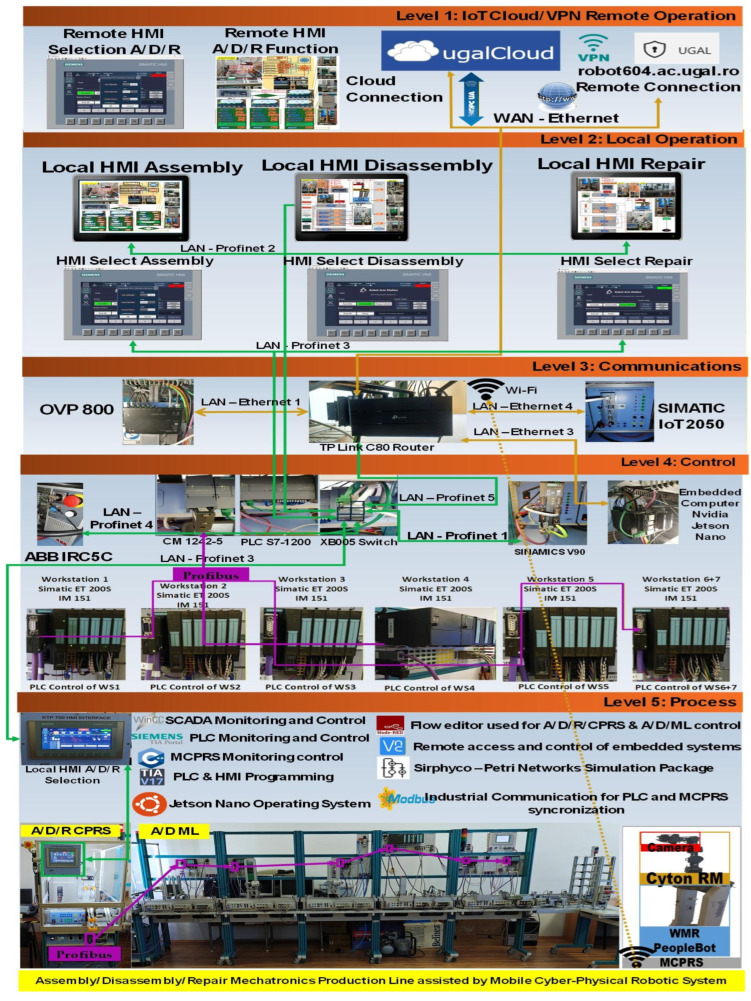
Global multilevel monitoring and control architecture.

This layered architecture, shown in Figure 7, ensures scalable integration of industrial automation components with cyber–physical and cloud-based services, enabling advanced functionalities such as remote monitoring, Digital Twin synchronization, and intelligent production management.

### 2.8. Human–Machine Interfaces

Operators interact with the system through both local and remote interfaces. Local interfaces include Siemens KTP700 HMI panel, an local SCADA monitoring systems.

These interfaces enable the operator to select assembly configurations, initiate production tasks, and monitor system status. The robotic subsystem is programmed and supervised using the ABB Flex Pendant, which provides direct access to robot programming and operational control.

**Figure 8:**
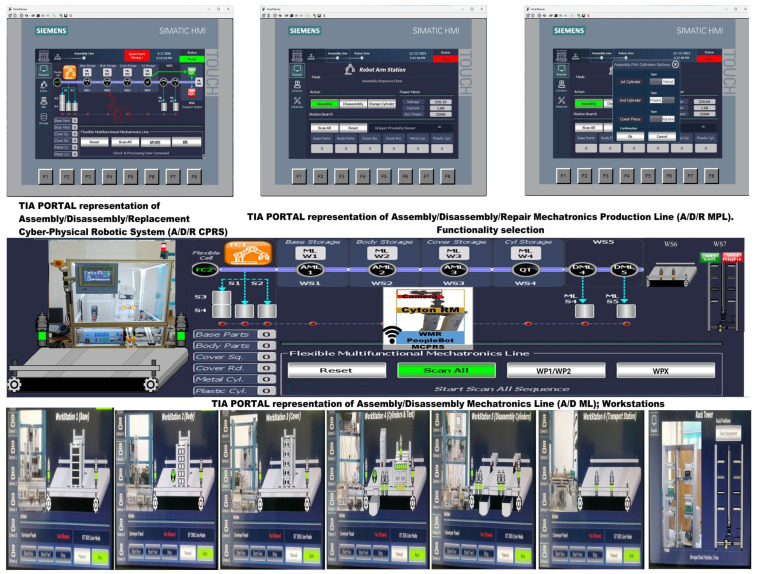
Human–Machine Interface and supervisory control of the A/D/R MPL.

Figure 7 and Figure 8 illustrate the operator interface and supervisory control and data acquisition (SCADA) environment of the A/D/R MPL, implemented in TIA Portal V17. The interface enables real-time monitoring and control of both subsystems: the A/D/R CPRS robotic cell and the A/D ML production line. Through this interface, the operator can select the type of workpiece to be assembled, initiate scanning and assembly sequences, monitor the availability of components in the warehouses (base, body, square cover, round cover, metal cylinders, and plastic cylinders), and supervise the overall production process. The interface also visualizes the status of each workstation and the flow of workpieces across the production line, facilitating real-time supervision and control of the cyber–physical manufacturing system.

**Figure 9:**
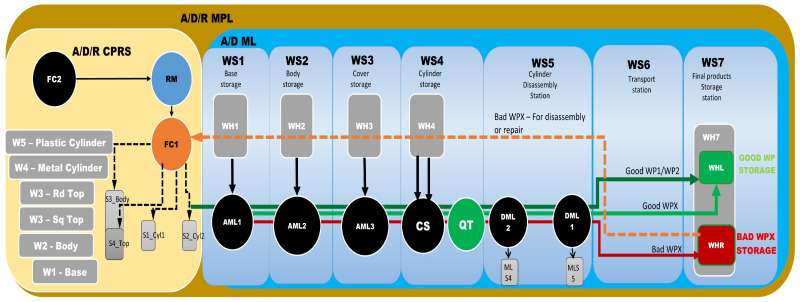
Stylized architecture of the integrated A/D/R MPL.

This Figure 9 presents the integrated stylized architecture of the complete A/D/R MPL, combining the A/D/R CPRS robotic cell (left) and the A/D ML production line (right) into a unified cyber–physical manufacturing system. In the CPRS, components are retrieved from the warehouses and assembled using the robotic manipulator within the flexible cell areas FC1 and FC2. The assembled workpieces are then transferred to the A/D ML. The workpieces WP1 and WP2, assembled within the CPRS, are assumed to be compliant products. Consequently, they bypass the quality testing station (QT) located at workstation WS4 of the A/D ML and are directly transported to the left storage rack (WHL) of workstation WS7, where compliant products are stored. WPs assembled on A/D ML, (named WPX) are quality tested and can be either fit (stored in WH), completely compromised or repairable and stored as completely damaged or repairable in WHR., where they pass through several workstations responsible for component feeding, quality testing, disassembly, and transportation. The QT station determines whether a workpiece is of acceptable quality. Good products WPX are directed to the WHL storage rack, while defective products are directed to the WHR storage rack for further disassembly or repair operations. The figure highlights the bidirectional interaction between the robotic cell and the mechatronic production line, enabling assembly, disassembly, repair, and component recovery within a flexible cyber–physical manufacturing environment.

Figure 1, Figure 2, Figure 3, Figure 4, Figure 5, Figure 6, Figure 7, Figure 8 and Figure 9 illustrate the physical architecture and operational integration of the Assembly/Disassembly/Repair Mechatronic Production Line (A/D/R MPL), including the robotic cyber–physical assembly cell, the modular mechatronic production line, the supervisory control interface, and the stylized integrated system architecture.

**Remark** **1.** *The workpieces WP1 and WP2, assembled within the A/D/R CPRS, are assumed to be compliant products. Consequently, they bypass the quality testing station (QT) located at workstation WS4 of A/D ML and are directly transported to the left storage rack (WHL) of workstation WS7, where compliant products are stored.*

### 2.9. Assumptions Regarding the Hardware Architecture

The operation of the A/D/R MPL, composed of the A/D/R CPRS and the A/D ML, and assisted by a MCPRS, depends on several factors such as operating modes, task execution times, system configuration, and the types of manufactured workpieces. The MCPRS assists the A/D/R MPL by performing manipulation and transportation operations of recovered components.

To enable coordinated operation and control of the entire cyber–physical manufacturing system, the following assumptions regarding the hardware architecture are considered.

**Assumption** **H1.** *The A/D/R MPL operates as a single-model production line. The system functions as a paced manufacturing line, meaning that transfers between workstations are synchronized. Furthermore, the production line is assumed to be deterministic, since the execution times of all operations are known a priori.*

**Assumption** **H2.** *The number of workstations composing the A/D/R MPL, including the A/D/R CPRS and the seven workstations WS1–WS7 of the A/D ML involved in assembly, disassembly, and repair operations, is predefined and remains constant during system operation.*

**Assumption** **H3.** *The workstations of the A/D/R MPL are arranged in a linear configuration, consisting of the A/D/R CPRS followed sequentially by WS1–WS7 of the A/D ML.*

**Assumption** **H4.** *The final storage station WS7 contains two storage racks. The left storage rack (WHL) is used for storing compliant products, while the right storage rack (WHR) stores workpieces that fail the quality test and therefore require disassembly or repair operations.*

**Assumption** **H5.** *A single MCPRS assists the A/D/R MPL. The MCPRS is a 2DW/1FW PeopleBot WMR equipped with a 7-DOF Cyton RM, used for picking, transporting, and depositing recovered components.*

**Assumption** **H6.** *An eye-in-hand MVSS is mounted on the end-effector of the Cyton RM, enabling visual perception and precise positioning during manipulation tasks.*

**Assumption** **H7.** *The motion of the MCPRS mobile platform is assumed to occur in an obstacle-free environment, and the platform moves with a constant translational velocity during transportation tasks.*

**Assumption** **H8.** *All disassembly operations and component replacement tasks are performed within the CPRS. Defective WPs are transferred to the CPRS, where the robotic manipulator executes the disassembly of components and the replacement of defective component when required.*

**Assumption** **H9.** *After disassembly operations performed in the CPRS, the recovered components are transferred through the dedicated output slides of the CPRS to the MCPRS. The MCPRS subsequently transports the recovered components and stores them in the corresponding component warehouses of the A/D ML for reuse.*

**Assumption** **H10.** *All operations of assembly, disassembly, and repair are executed in a synchronized manner through coordination signals exchanged between PLCs operating in a master–slave configuration. These synchronization signals ensure coordinated operation among the three major subsystems of the MPL, CPRS, ML, and the MCPRS.*

**Assumption** **H11.** *The communication infrastructure of the A/D/R MPL ensures reliable and deterministic data exchange between all subsystems. Communication among the A/D/R CPRS, the A/D ML, and the MCPRS is achieved through industrial communication networks such as PROFIBUS DP, LAN-PROFINET, LAN Ethernet, and WAN Ethernet, while higher-level monitoring and supervisory control are supported through OPC-UA communication services. Assumptions H12. Network latency is assumed to be sufficiently small so that communication delays do not affect the synchronization of operations coordinated by the PLC-based distributed control architecture.*

**Remark** **2.** *The assumptions H1-H12 establish the structural, operational, and communication constraints of the A/D/R MPL and provide the foundation for the formal modelling and simulation of the system. In particular, the coexistence of discrete-event dynamics, related to workstation operations, task sequencing, and continuous dynamics, associated with the motion of the MCPRS mobile platform, motivates the use of SHPN for system modelling.*

The above assumptions were primarily adopted to simplify the modelling, synchronization analysis, and real-time implementation of the proposed A/D/R MPL assisted by dual CPRS within a controlled laboratory-scale experimental environment. In particular, assumptions related to obstacle-free navigation, negligible communication delays, ideal accessibility conditions, and reduced equipment uncertainties were considered appropriate for validating the proposed cyber–physical control architecture, SHPN synchronization framework, DT integration, and AI-assisted control strategies under reproducible experimental conditions. The assumptions are mainly applicable to the controlled experimental platform used for implementation and validation, and do not fully represent all constraints encountered in large-scale industrial deployment scenarios. In actual industrial environments, additional factors such as dynamic obstacle avoidance, variable communication latency, packet loss, actuator wear, sensor degradation, equipment calibration errors, safety protection constraints, human–robot interaction, and cybersecurity threats must also be considered.

Within this framework, synchronized timed Petri Nets (STPN) are used to model the discrete operations executed at the workstations of the A/D/R CPRS and the A/D ML, while Continuous Petri Nets (CPN) describe the continuous motion and positioning of the MCPRS. The synchronization signals exchanged between the PLCs enable coordinated interaction between these discrete and continuous processes. Consequently, the SHPN model provides an appropriate formal representation of the integrated cyber–physical manufacturing system and allows simulation and analysis of the assembly, disassembly, repair, and component recovery workflows before the implementation of real-time control.

### 2.10. AI Hardware Integration and Intelligent Edge–Cloud Architecture

To enhance autonomy, adaptability, and sustainability of the A/D/R MPL assisted by MCPRS, an AI-enabled edge–cloud architecture is integrated into the hardware layer. The intelligence layer combines embedded computing, industrial IoT gateways, sensor networks, and PLC-based deterministic control.

- Edge AI Processing Unit—NVIDIA Jetson Nano. The NVIDIA Jetson Nano embedded computer serves as the local AI edge processor. It is connected via Ethernet (LAN-ETH4) to the industrial router and communicates with: Siemens S7-1200 PLC (Profinet), SIMATIC IoT2050 gateway, Vision system (USB camera), ABB IRC5 controller (via LAN interface), and MCPRS subsystem (Wi-Fi synchronization).
  As functional responsibilities, Jetson-Nano performs:
  1. Machine learning inference for WP classification (WP1/WP2), material recognition (metal/plastic cylinder), fault detection, and WPX quality assessment,
  2. Reinforcement learning-based adaptation for adjustment of robot insertion parameters, adaptive gripping force tuning, and recovery strategy selection,
  3. Fuzzy decision supervision for handling uncertain sensor signals and soft transition between acceptable/repairable/reject states,
  4. Fractional-order learning tuning for smoothing of adaptation signals and memory-based learning for repetitive assembly cycles.

The Jetson-Nano operates as an edge intelligence layer, ensuring real-time decision-making without cloud latency.

- Industrial IoT gateway—SIMATIC IoT2050. The SIMATIC IoT2050 functions as a secure data acquisition and preprocessing node having the following roles: aggregation of inductive sensor signals, position signals, quality test data (QT-WS4), cylinder verification results, OPC-UA communication to cloud, secure VPN data forwarding. and edge-to-cloud synchronization. The SIMATIC IoT2050 gateway decouples deterministic PLC control from non-deterministic AI processes.
- Sensor integration for AI learning. The AI layer utilizes multi-source sensor data:
  1. Inductive sensors for: base coding detection (6-bit ID), position verification, and material presence detection,
  2. Vision system (Eye-in-Hand MVSS) with high-definition Logitech camera for: object pose estimation, alignment correction, and quality verification,
  3. Cylinder test station (WS4) for material detection, pass/fail signal, and mechanical verification,
  4. Robot internal sensors for joint position, velocity, torque estimation, and gripper state.

All sensor data are fused at the edge for real-time decision-making.

### 2.11. Digital Twin Architecture of the A/D/R MPL Assisted by Dual CPRS

The proposed system is based on a DT architecture that integrates the physical manufacturing environment, the cyber control infrastructure, and the virtual world used for monitoring, planning, and simulation. The DT enables bidirectional interaction between the real production system and its virtual representation, allowing real-time supervision, predictive analysis, and decision support for the A/D/R MPL assisted by the MCPRS. The architecture is structured into three main layers, physical layer, cyber layer, and virtual world layer.

- Physical layer. The physical layer represents the real manufacturing environment and includes the hardware infrastructure of the A/D/R MPL and the MCPRS. The A/D/R MPL integrates the CPRS, the A/D ML, and the associated storage stations and workstations responsible for assembly, disassembly, inspection, and repair operations. Within this environment, the ABB IRM performs assembly and disassembly operations in the CPRS, while the Cyton 1500 RM, mounted on the PeopleBot WMR, forms the MCPRS responsible for transporting recovered components and assisting disassembly and repair operations. The MCPRS is equipped with an eye-in-hand MVSS, allowing accurate positioning during pick-and-place tasks. The physical layer also includes the PLC-based automation infrastructure, sensors, actuators, conveyor systems, and warehouse modules that enable the execution of the assembly, disassembly, and repair processes.
- Cyber layer. The cyber layer ensures communication, control, and data exchange between the physical system and its virtual counterpart. This layer integrates industrial control and networking technologies, including:
  1. PLCs responsible for real-time control of the MPL workstations,
  2. industrial communication networks that connect the MPL modules and the MCPRS,
  3. SCADA and HMI systems used for monitoring and operator interaction,
  4. IoT gateways and communication interfaces that enable data exchange between the local system and remote supervisory platforms.

Within this layer, synchronization signals generated by PLCs coordinate the interaction between the MPL processes and MCPRS actions. These signals allow the execution of distributed tasks while maintaining consistent system states between the physical and virtual environments.

- Virtual world layer. The virtual world layer represents the digital counterpart of the A/D/R MPL assisted by MCPRS and provides tools for task planning, monitoring, simulation, and decision support. This layer integrates two complementary technologies:
  1. AR implemented with Node-RED [48];
  2. VR implemented through SHPN modelling and simulation.

The AR environment enables the visualization and management of production tasks in real time, while the VR environment models the system behaviour and allows predictive analysis through simulation.

### 2.12. Conclusions About the Digital Twin and Control Architectures

The integration of AR-based task planning and VR-based SHPN simulation within the Digital Twin framework provides a comprehensive environment for supervising and optimizing the A/D/R MPL assisted by MCPRS. The virtual world enables real-time visualization of system operations, predictive evaluation of workflows, and validation of control strategies before their deployment in the physical system. By synchronizing the discrete manufacturing processes of the MPL with the continuous motion of the MCPRS, the SHPN model supports the development of a distributed control architecture based on synchronization signals generated by the PLCs. This approach contributes to the realization of Industry 4.0 manufacturing systems, where cyber–physical integration, Digital Twins, and advanced simulation tools enable intelligent automation and flexible production. Furthermore, the inclusion of mobile robotic assistance and component recovery mechanisms aligns the proposed architecture with the principles of Industry 5.0, emphasizing sustainability, resource efficiency, and human-centred supervision of complex manufacturing systems.

## 3. The Digital Twin Virtual Layer of the A/D/R MPL Assisted by the Dual CPRS

The virtual layer represents the digital part of the A/D/R MPL composed of the A/D/R CPRS, and the A/D ML, assisted by the MCPRS. The virtual layer integrates task planning, system monitoring, workflow simulation, and synchronization mechanisms, enabling the analysis and optimization of the manufacturing process before real-time deployment. The approach combines AR for interactive task planning and VR for formal modelling and simulation of the production workflow.

### 3.1. AR and VR as Components of the DT Virtual Layer

The virtual layer, representing the DT of the A/D/R MPL assisted by the dual CPRS, integrates two complementary components:

- AR implemented using Node-RED, which supports task planning, monitoring, and interaction with the production system,
- VR implemented using SHPN simulated with the Sirphyco package, which models the workflow and system dynamics [49].

### 3.2. AR-Based Task Planning Using Node-RED

**Figure 10:**
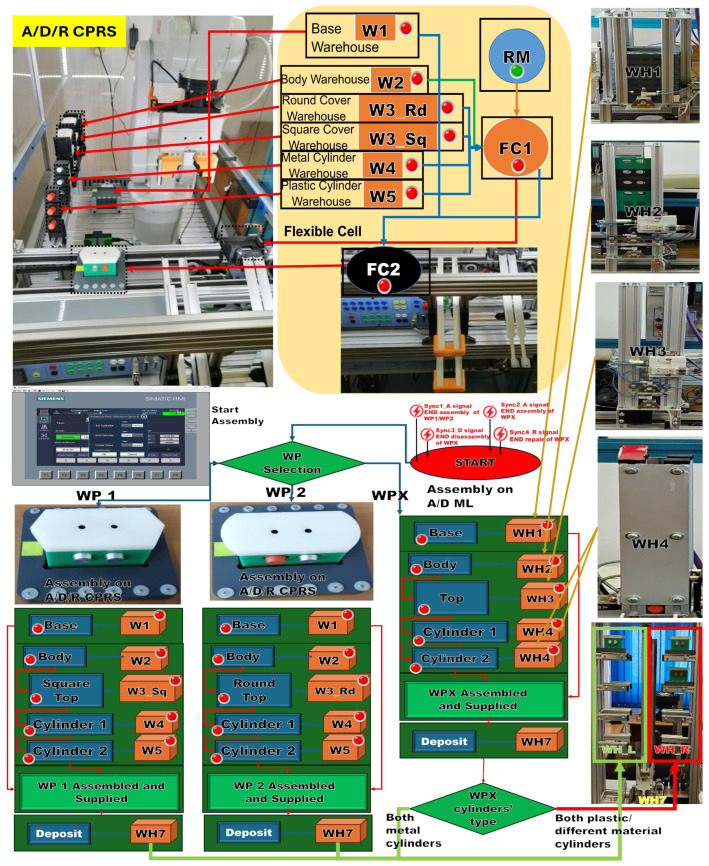
Node-RED task planning as AR for assembly in A/D/R MPL (WP1/WP2 in CPRS and WPX in A/D ML).

**Figure 11:**
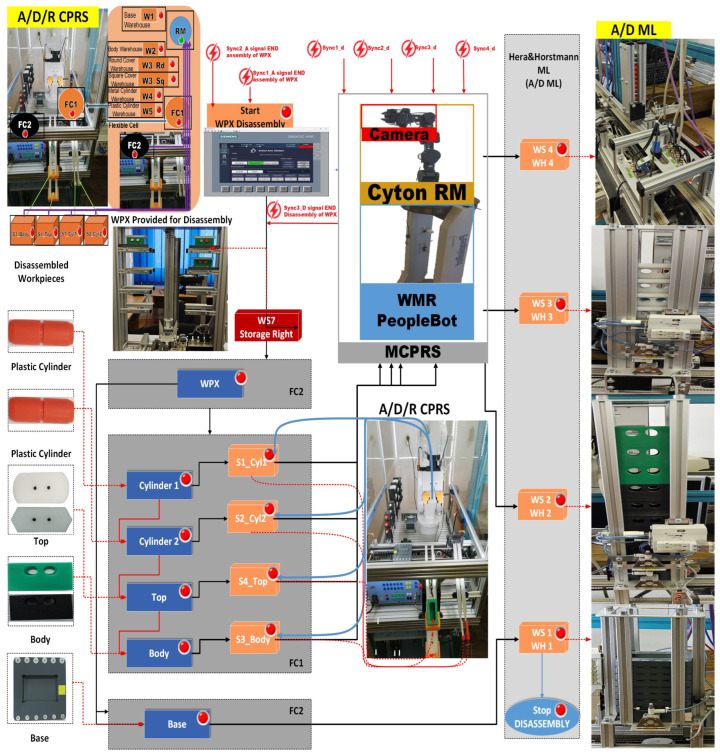
Node-RED task planning as AR for disassembly in A/D/R MPL assisted by MCPRS.

**Figure 12:**
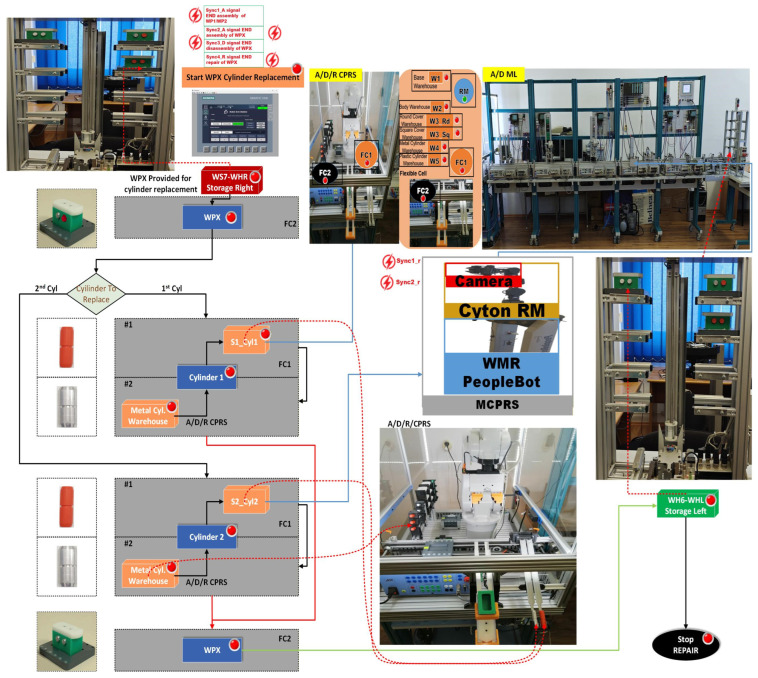
Node-RED task planning as AR for repair in A/D/R MPL assisted by MCPRS.

The task planning diagrams presented in Figure 10, for assembly, Figure 11, for disassembly and Figure 12, for repair, describe the complete set of functionalities of the MPL, namely assembly, disassembly, and repair, respectively. These figures illustrate the coordinated interaction between the A/D/R MPL and the MCPRS, including component handling, storage, recovery, and replacement.

Node-RED is used to implement the AR component of the virtual world, enabling the development of interactive dashboards and task management interfaces. Through this platform, users can design automation workflows, monitor task execution in real time, and receive alerts or updates regarding the system status. Node-RED simplifies the programming of automation flows and integrates efficiently with the SCADA/HMI infrastructure. In the proposed architecture, task planning is implemented as an AR workflow composed of Node-RED functions. These workflows are deployed on two HMIs:

1. MPL operation HMI, responsible for monitoring and controlling the operations executed on the A/D/R CPRS and A/D ML,
2. MCPRS assistance HMI, dedicated to supervising the operations performed by the mobile robotic system, including component transport and storage.

The tasks defined in the organizational workflow are executed synchronously using PLC synchronization signals. Each task execution generates a monitoring signal, which is visualized in the organizational chart by activating a corresponding indicator. The AR interface enables the user to interact visually with the production system and observe the execution of operations in real time. In this environment, the operator can monitor the activities of the MPL workstations, and the actions performed by the MCPRS, including the transport, pick-and-place manipulation, and storage of workpiece components.

The AR interface also allows the user to evaluate alternative task sequences and workflow modifications to predict system performance. Within the AR interface, each task in the organizational workflow corresponds to a Node-RED functional block. The execution of a task is triggered through synchronization signals generated by the PLCs, ensuring coordinated operation among the subsystems. The progress of the workflow is visually represented through status indicators or “spotlights”, which illuminate when the corresponding monitoring signals are activated.

### 3.3. VR-Based Modelling and Simulation Using SHPN

The VR component of the virtual world is implemented through SHPN and simulated using the Sirphyco package. This modelling framework enables the representation of workflow evolution, resource dependencies, and synchronization between discrete and continuous processes. In this approach:

STPN describe the discrete operations executed at MPL workstations, including assembly, disassembly, and repair processes.

Continuous Petri Nets (CPN) represent the continuous displacement of the MCPRS, including trajectory generation, movement speed, and interaction with the production system.

**Figure 13:**
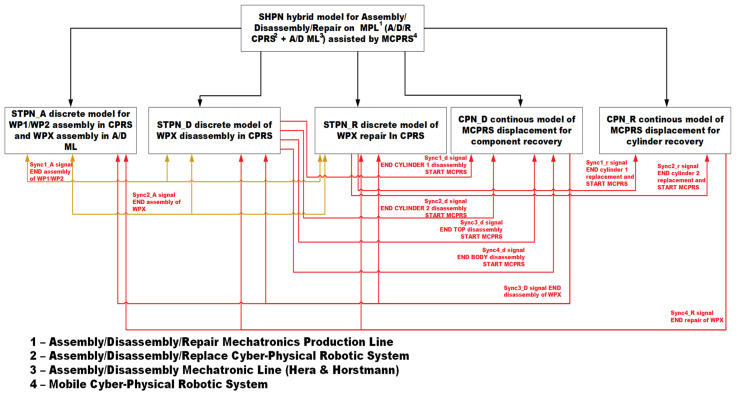
SHPN structure of A/D/R MPL assisted by dual CPRS.

The SHPN model captures both the discrete-event dynamics of the manufacturing process and the continuous dynamics associated with mobile robot motion. The SHPN models are simulated using the Sirphyco simulation package, which allows the analysis of system behaviour, synchronization constraints, and resource allocation before real-time implementation. The modelling framework includes three main types of Petri Net models, as is shown in Figure 13:

Synchronised Timed Petri Net (STPN). STPN is used to model the assembly operations performed on the production line. As illustrated in the system architecture figures, two assembly processes are considered: assembly of WP1 and WP2 in the A/D/R CPRS, and assembly of WPX in the A/D ML.

The A/D/R CPRS contains several component warehouses: W1—Base warehouse, W2—Body warehouse, W3_Rd—Round Top (Cover) warehouse, W3_Sq—Square Top (Cover) warehouse, W4—Metal cylinder warehouse, and W5—Plastic cylinder warehouse.

Using the robotic manipulator, components are sequentially retrieved from these warehouses and assembled in the flexible cell (FC1) before the workpiece is transferred to the conveyor system FC2.

Two types of products are assembled in the CPRS:

- WP1, characterized by a Top (Cover) with square edges,
- WP2, characterized by a Top (Cover) with round edges.

After assembly, these workpieces are transported along the A/D ML conveyor and stored directly in the left warehouse (WHL) at workstation WS7, since they are considered compliant products and do not require quality testing at WS4. In contrast, WPX products are assembled in the A/D ML, using the component warehouses of the mechatronics line.

1. SHPN models for disassembly and repair (SHPN_D and SHPN_R). The SHPN framework is used to model disassembly and repair processes, which apply only to WPX products. After assembly on the A/D ML, WPX products are subjected to a quality test at workstation WS4. Depending on the detected cylinder configuration, three cases are possible:
  - Both cylinders are metallic. The product is considered compliant and is stored in the WHL warehouse at WS7.
  - Both cylinders are plastic. The product is classified as defective and is stored in the right warehouse (WHR). These products are subsequently transferred to the A/D/R CPRS for disassembly, enabling component recovery.
  - Mixed cylinders (plastic and metal). The product requires repair through cylinder replacement. It is therefore sent to the A/D/R CPRS, where the plastic cylinder is replaced with a metallic one.
2. CPN for MCPRS motion. The continuous dynamics of the MCPRS are modelled using Continuous Petri Nets (CPN). Two CPN models are used:
  - CPN_D, representing the displacement of the MCPRS during component recovery following disassembly operations,
  - CPN_R, representing MCPRS movements during repair operations involving cylinder replacement.
  - The MCPRS performs the following functions:
  - Retrieving disassembled components from the CPRS output slides,
  - Transporting the components to the component warehouses of the A/D ML,
  - Storing reusable components for future assembly operations.

### 3.4. Synchronization Between Models

The synchronization signals shown in Figure 13 represent the coordination mechanism between the discrete-event SHPN/STPN models of the A/D/R MPL, the A/D/Replacement CPRS, and MCPRS. These synchronization events ensure deterministic sequencing and safe interaction among assembly, disassembly, repair, warehouse transport, and MCPRS displacement operations. The synchronous signals are not simple status indicators, instead, they act as global event-triggering conditions enabling transitions between the major operational phases of the unified SHPN framework. The complete synchronization logic is governed by four principal synchronization signals, which correspond respectively to:

- Sync1_A—END assembly of WP1/WP2 in the A/D/R CPRS,
- Sync2_A—END assembly of WPX in the A/D ML,
- Sync3_D—END disassembly of WPX, including MCPRS displacement completion,
- Sync4_R—END repair of WPX, including MCPRS return displacement completion.

When the assembly of WP1/WP2 is fully completed, the synchronization signal Sync1_A is generated. This signal has two simultaneous functions:

- It confirms the completion of local assembly operations inside the CPRS.
- It enables the start of the WPX assembly process in the A/D ML.

After WPX assembly is completed in the A/D ML, the synchronization signal Sync2_A is generated. This event indicates that the complete workpiece is available for subsequent disassembly or repair operations. Therefore, the assembly sequence follows the timing logic: WP1/WP2 Assembly→Sync1A→WPX Assembly→Sync2A.

The disassembly process is activated only after the WPX assembly has been completed and both cylinders are plastic.

During disassembly, several intermediate synchronization signals are generated:

- Sync1_d: END disassembly of Cylinder 1 and START MCPRS,
- Sync2_d: END disassembly of Cylinder 2 and START MCPRS,
- Sync3_d: END disassembly of Top component and START MCPRS,
- Sync4_d: END disassembly of Body component and START MCPRS.

These local synchronization events trigger the continuous MCPRS displacement model represented by the Continuous Petri Net (CPN_D). The global disassembly phase terminates only after:

- all component disassembly operations are completed;
- MCPRS transportation missions are completed.

At this moment, the global synchronization signal Sync3_D is generated.

Sync3_D: END WPX disassembly and MCPRS in parking position (point O).

Therefore, Disassembly + MCPRS displacement → Sync3D.

The repair process is activated only after the WPX assembly has been completed and both cylinders are of different material. During repair, the MCPRS, after cylinder replacement, transports recovered parts to WH4 of WS4. Intermediate synchronization signals include:

- Sync1_r: END replacement of Cylinder 1 and START MCPRS,
- Sync2_r: END replacement of Cylinder 2 and START MCPRS.

These signals activate the continuous MCPRS displacement model represented by CPN_R. The repair phase terminates only when:

- all replacement operations are completed,
- all MCPRS recovery/transportation tasks are finalized.

At this moment, the global synchronization signal Sync4_R is generated.

Sync4_R: END WPX repair and MCPRS in parking position (point O).

Then the global synchronization signal Sync4_R is generated, indicating Repair + MCPRS return displacement → Sync4_R.

This global synchronization guarantees:

- Deterministic sequencing,
- Deadlock-free operation,
- Synchronization between discrete-event and continuous dynamics,
- Safe cyber–physical coordination,
- Consistency between DT and real system execution.

The synchronization mechanism therefore acts as a supervisory event layer linking:

- STPN assembly models,
- SHPN disassembly/repair models,
- Continuous MCPRS displacement models,
- Node-RED orchestration,
- OPC-UA communication,
- Cloud-based monitoring/control infrastructure.

Through this synchronized modelling approach, the DT can represent the complete manufacturing workflow, including assembly operations, disassembly processes, repair procedures, and mobile robot transportation tasks. Thus, the integrated system exhibits both discrete and continuous dynamics, resulting in a hybrid model that captures the behaviour of the entire system. The discrete dynamics correspond to assembly, disassembly, and repair operations performed on the MPL, whereas the continuous dynamics correspond to the MCPRS displacement during pick-and-place operations and component recovery.

### 3.5. Role of SHPN in System Simulation and Control Design

The SHPN models proposed for the A/D/R MPL assisted by MCPRS are essential for system simulation and represent the stage preceding the implementation of real-time control. Through simulation, the evolution of the integrated system can be monitored in the state space, as transitions occur between system states according to the defined operational logic. The input parameters used in the SHPN models include:

- The sequence and duration of operations on the A/D/R MPL,
- The travel distances and motion durations of the MCPRS,
- Manipulation times associated with each pick-and-place operation,
- The positioning times of the Cyton 1500 robotic manipulator when retrieving components from disassembly locations and storing them in the corresponding warehouses.

Accurate positioning times represent a significant uncertainty due to mechanical constraints and environmental variations that may affect real-time control performance. To address this issue, the system integrates a mobile eye-in-hand visual servoing system, which improves positioning accuracy during component manipulation. The SHPN simulations provide decision-support information for designing the control architecture of the integrated system.

### 3.6. Structure of the SHPN Models

In the SHPN representation:

- Red places correspond to control states associated with decision-making functions and trigger transitions when they receive tokens.
- Brown or Gray places represent pick-and-place manipulation actions, receiving tokens upon completion of the corresponding transitions.
- Yellow places represent monitoring states, which receive tokens after the completion of handling, transport, or processing operations.
- Green places correspond to final delivery states, indicating the completion of assembly or cylinder replacement operations.
- Red synchronization places represent synchronization signals, which receive tokens when a specific functionality of the MPL or an MCPRS action has been completed.

The synchronization signals coordinate the interaction between the MPL processes and the MCPRS operations, ensuring that the execution of subsequent tasks starts only after the required preceding operations have been completed.

### 3.7. DT Virtual Layer Regarding Assembly

1. Assumptions regarding assembly. To formally describe the assembly workflow within the A/D/R MPL, a set of assumptions related to product types, operational conditions, and quality evaluation criteria are defined.

**Assumption** **A1.** *Two types of workpieces are assembled in the A/D/R CPRS) using the ABB industrial robotic manipulator (IRM): WP1, characterized by a top with square edges, WP2, characterized by a top with round edges. In addition, workpieces WPX (X = 1 or 2) are assembled on the Assembly/Disassembly Mechatronic Line (A/D ML).*

**Assumption** **A2.** *All operational parameters of the assembly process are known a priori, including task sequences, component availability, and execution durations.*

**Assumption** **A3.** *Workpieces WP1 and WP2, assembled in the CPRS, are considered compliant products. Therefore, they bypass the quality testing stage and are directly stored in the left warehouse (WHL) of workstation WS7 on the A/D ML.*

**Assumption** **A4.** *A WPX workpiece assembled on the A/D ML is considered compliant if, after the quality test performed at WS4, it contains two metal cylinders. Such products are stored in the WHL of WS7.*

**Assumption** **A5.** *By convention, a WPX workpiece fails the quality test if it contains: two plastic cylinders, or cylinders made of different materials (one plastic and one metal).*

**Assumption** **A6.** *A WPX workpiece that fails the quality test is stored in the right warehouse (WHR) of WS7, where it is subsequently considered for disassembly or repair operations in the A/D/R CPRS.*

2. Task assignment, planning, and synchronization. The assembly of WP1 and WP2 is performed by the ABB IRM located in the A/D/R CPRS. The robot retrieves components sequentially from the CPRS warehouses in the following order: base, body, top element (square or round), two cylinders (metal or plastic). Initially, the base is positioned on the conveyor belt at FC2. The remaining components are assembled at a separate location within the flexible cell (FC1). After completing the upper structure, the ABB IRM transfers the assembled subassembly onto the base located on FC2. Once the assembly is completed, the workpieces WP1 and WP2 are transported along the A/D ML and directly stored in the WHL of WS7, since they are considered compliant and do not require quality testing.

The system is supervised through a Human–Machine Interface (HMI), which provides a graphical user interface (GUI) enabling the operator to configure the assembly process. The user can select top geometry (square or round) and cylinder material (metal or plastic).

Due to the controlled assembly process in the CPRS, WP1 and WP2 are always considered high-quality products.

In contrast, the WPX workpieces, assembled on the A/D ML, are generated through an automated process with random cylinder selection. Therefore, these products are subjected to a quality test at workstation WS4.

All assembly operations within the A/D/R MPL are coordinated through PLC-based synchronization signals, ensuring proper interaction between: A/D/R CPRS, and A/D ML.

3. Virtual digital layer for the assembly (unified formalism), modelling approach, and justification.

**Figure 14:**
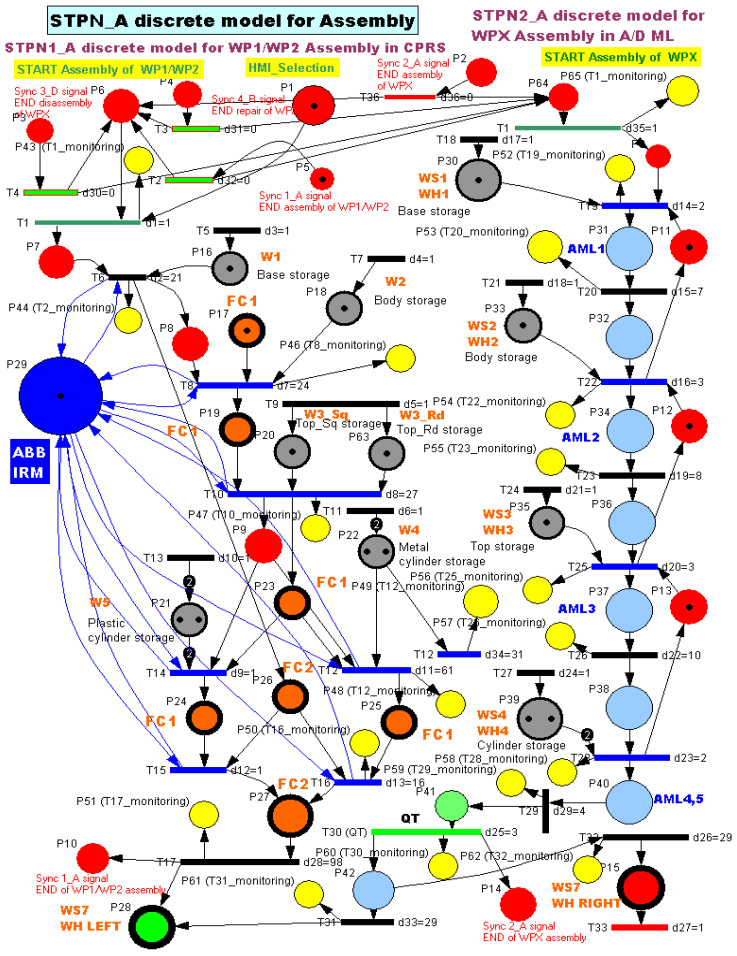
STPN model as VR for assembly of WP1/WP2 in CPRS and WPX in A/D ML.

The assembly process of workpieces WP1, WP2, and WPX is modelled using a Synchronized Timed Petri Net (STPN_A). The STPN_A model is shown in Figure 14.

The use of STPN is justified by the parallel operation of two subsystems: the A/D/R CPRS (assembly of WP1/WP2), the A/D ML (assembly and storage of WPX), and the necessity of synchronization between assembly, disassembly, repair processes, and the integration of timing constraints (task durations, transport delays).

The unified STPN_A model definition consists of

(1) $$N_{STPN\text{\_}A}=\left(P,T,Pre,Post,M_{0},\Sigma ,Sync,D\right),$$

where

- P: set of places;
- T: set of transitions;
- $Pre,Post:P\times T\to R^{+}$ are the input/output incidence functions;
- $M_{0}$: initial marking;
- Σ: synchronization signals;
- Sync⊆T×Σ: synchronization mapping;
- $D:T\to R^{+}$: transition firing delays.

The structure of the model consists of

Places

(2) $$P=P_{A}^{CPRS}\cup P_{A}^{ML}\cup P_{mon},$$

where

- $P_{A}^{CPRS}$: assembly states for WP1/WP2;
- $P_{A}^{ML}$: assembly states for WPX;
- $P_{mon}$: monitoring and synchronization places.

Transitions

(3) $$T=T_{A}^{CPRS}\cup T_{A}^{ML},$$

where:

- $T_{A}^{CPRS}$: assembly operations (ABB IRM, FC1, FC2),
- $T_{A}^{ML}$: transport, storage, and quality test operations.

Marking vector (unified form) consists of

(4) $$M(t)=M_{d}(t),$$

Since STPN_A is purely discrete,

(5) $$M_{d}(t)\in N^{|P|}.$$

Timing dynamics. Each transition $t_{i}\in T$ is associated with a delay, $d_{i}$.

(6) $$t_{i}\to \mathrm{fires}\;\mathrm{after}\;d_{i},$$

Thus, the marking evolution is as follows:

(7) $$M(t^{+})=M(t)+(Post-Pre)\cdot \sigma (t),$$

where $\sigma \left(t\right)$ represents the firing sequence under timing constraints.

Synchronization (Global consistency). The same global synchronization set is used:

(8) Σ={Sync1_A,Sync2_A,Sync3_D,Sync4_D},

with

- Sync1_A: END assembly of WP1/WP2, in CPRS,
- Sync2_A: END assembly of WPX, in A/D ML,
- Sync3_D: END disassembly of WPX and MCPRS in parking position (point O),
- Sync4_R: END repair of WPX and MCPRS in parking position (point O).

Synchronization rule (unified across all sections):

(9) $${Sync_{k}\to t}_{i}\to t_{j},$$

ensuring and coordination between CPRS and A/D ML and correct sequencing between assembly, disassembly, and repair.

**Figure 15:**
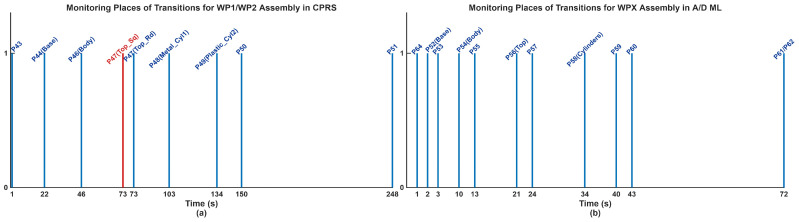
STPN_A model of the assembly process in the A/D/R MPL. (**a**) Evolution of monitoring places $M_{d}\left(t\right)$ for the assembly of WP1/WP2 in the A/D/R CPRS. (**b**) Evolution of monitoring places $M_{d}\left(t\right)$ for the assembly of WPX in the A/D ML.

Simulation results and interpretation. Simulation in Sirphyco validates the STPN_A model, Figure 15 [50]:

- Parallel synchronization between CPRS and A/D ML is ensured,
- No deadlocks or conflicts occur,
- Timing constraints are respected.

### 3.8. DT Virtual Layer Regarding Disassembling

1. Assumptions regarding disassembly. To formally describe the disassembly workflow within the A/D/R MPL framework, a set of assumptions is defined related to the type of WP that is disassembled according to the quality assessment criteria and the way in which the disassembled components are recovered and revalued.

**Assumption** **D1.** *All conditions and parameters of the disassembly process are initially known, including task sequences and execution durations.*

**Assumption** **D2.** *A workpiece WPX is considered to have failed the quality test and becomes eligible for disassembly if it contains two plastic cylinders, according to the adopted quality convention.*

**Assumption** **D3.** *A WPX that fails the quality test is stored in the right warehouse (WHR) of workstation WS7, from where the disassembly process is subsequently initiated.*

**Assumption** **D4.** *All disassembly operations of WPX are executed within the A/D/R CPRS, using the ABB industrial robotic manipulator (IRM).*

**Assumption** **D5.** *The disassembly process is performed in a predefined sequence: cylinder 1 (left), cylinder 2 (right), top, and body, executed at the flexible cell FC1, with components guided through dedicated mechanical troughs.*

**Assumption** **D6.** *The Mobile Cyber–Physical Robotic System (MCPRS) retrieves the disassembled components sequentially (cylinder 1, cylinder 2, body, and top) and transports them to the corresponding storage warehouses within the A/D ML.*

**Assumption** **D7.** *The base component is transported back to warehouse WH1 located on the A/D ML, where a pneumatic mechanism ensures its proper storage.*

**Figure 16:**
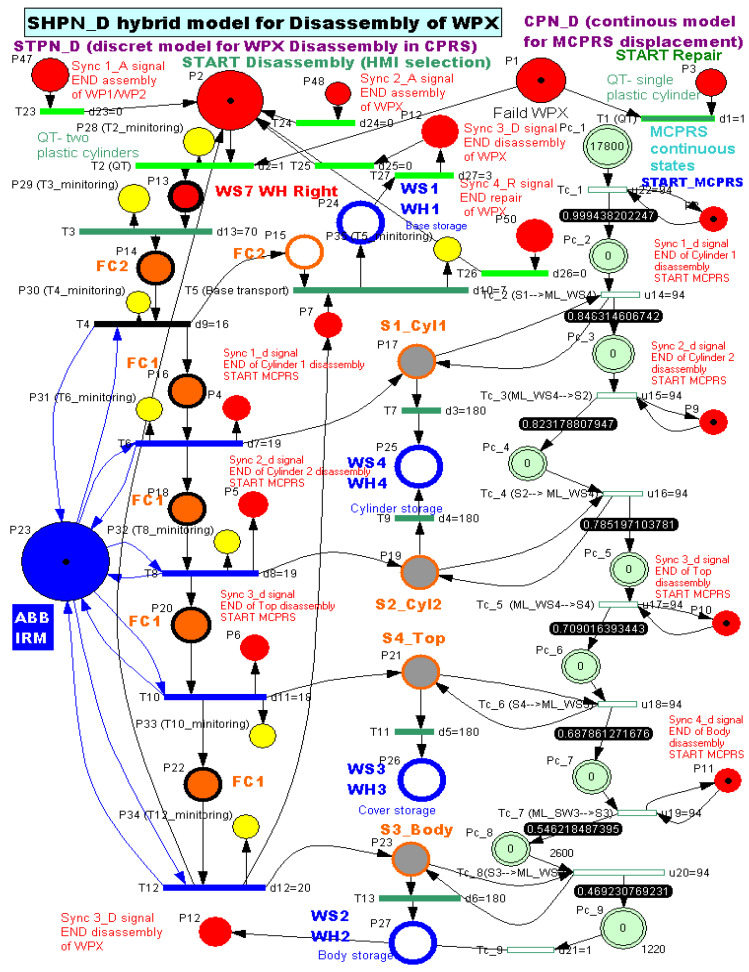
SHPN_D model as VR for disassembly of WPX in A/D/R MPL.

2. Task assignment, planning, and synchronization. The disassembly process, shown in Figure 11, is carried out in the A/D/R CPRS by the ABB IRM, following a predefined and synchronized sequence to ensure efficient component recovery. Initially the workpiece WPX, previously identified as defective and stored in WHR o f WS7, is transferred to the CPRS for disassembly. The ABB IRM performs the disassembly operations at the flexible cell FC1, respecting the following order: removal of cylinder 1 (left), removal of cylinder 2 (right), removal of the top component and removal of the body component. Each extracted component is directed via mechanical troughs toward predefined collection points. The base component is separately transported back to WH1 on the A/D ML, where it is automatically stored using a pneumatic actuator. Following the disassembly stage, the MCPRS performs the component recovery and redistribution tasks. It sequentially picks up each component and transports it to the appropriate storage warehouse within the A/D ML (e.g., WH1–WH4), according to its type. To ensure high precision during manipulation, the MCPRS employs an eye-in-hand MVSS mounted on the robotic manipulator. This enables accurate positioning during pick-and-place operations despite uncertainties in the environment. The entire workflow is coordinated through PLCs-based synchronization signals, ensuring proper interaction between the CPRS (disassembly operations), and the MCPRS (transport and storage operations). The corresponding task planning and synchronization diagrams (as illustrated in the provided figures) describe the logical sequence of operations and signal-based coordination. The disassembly functionality is intrinsically linked to component recovery and reuse, forming a key element of the circular manufacturing strategy implemented in the system.
3. Synchronization signals. The coordination between discrete (robotic operations) and continuous (MCPRS motion) dynamics is ensured through:
  Sync1_d: END Cylinder 1 disassembly and START MCPRS,
  Sync2_d: END Cylinder 2 disassembly and START MCPRS,
  Sync3_d: END Top (Cover) disassembly and START MCPRS,
  Sync4_d: END Body disassembly and START MCPRS,
  Sync3_D: END WPX disassembly and MCPRS in parking position (point O).
4. SHPN model, formalism, and simulation. The disassembly process is modelled using SHPN, denoted as SHPN_D, shown in Figure 16, derived from the task planning and synchronization diagrams. The SHPN_D model is represented as an oriented graph that integrates both discrete-event dynamics and continuous-time dynamics, providing a comprehensive description of the system behaviour.

The discrete component (STPN-based) models the disassembly operations executed by the ABB IRM within the CPRS (component removal sequence at FC1). The continuous component (CPN-based) models the MCPRS displacement, including navigation, component pickup, transportation, and storage. Through this integration, the model captures the hybrid nature of the system, where discrete events govern manufacturing operations, and continuous dynamics describe robotic motion and interaction with the environment. The SHPN_D model enables: formal representation of task synchronization, analysis of resource allocation and timing constraints, and simulation of the disassembly workflow prior to real-time implementation. During simulation, the system evolution can be tracked in the state space, ensuring consistency between task execution, synchronization signals, and resource availability. Furthermore, the SHPN framework supports the evaluation of system performance in terms of: operational efficiency, coordination between CPRS and MCPRS, and effectiveness of component recovery processes. This modelling approach constitutes a critical step toward the implementation of real-time control strategies within the Digital Twin framework, enabling seamless interaction between the physical system and its virtual counterpart.

**Figure 17:**
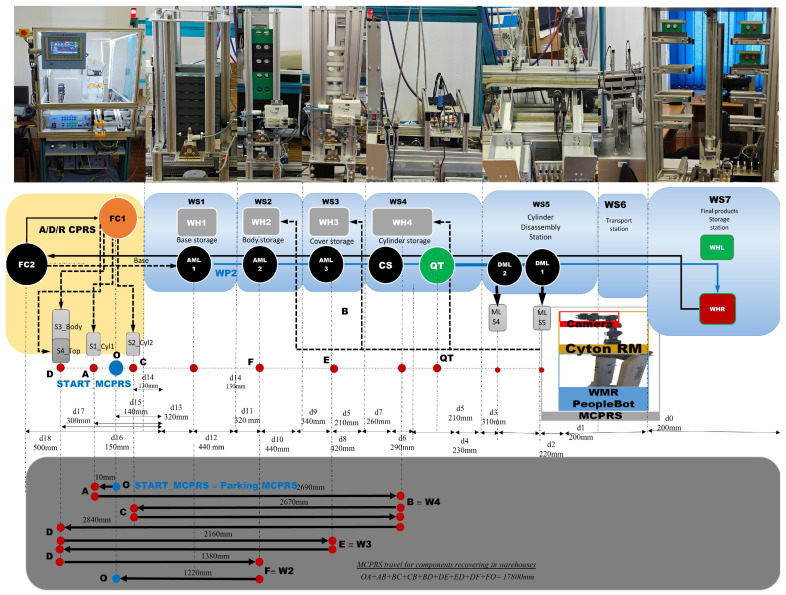
Distances travelled by MCPRS to recover components during WPX disassembly. O→A (S1): pickup of the first cylinder, A→B (WH4/WS4): storage of the first cylinder, B→C (S2): pickup of the second cylinder, C→B: storage of the second cylinder, B→D (S4): pickup of the top (cover), D→E (WH3/WS3):storage of the top, E→D (S3), pickup of the body, D→F (WH2/WS2): storage of the body, F→O: return to parking position. The pallet (base) handling is not performed by the MCPRS; it is ensured by conveyor systems and pneumatic actuators within the A/D/R CPRS and A/D ML.

**Figure 18:**
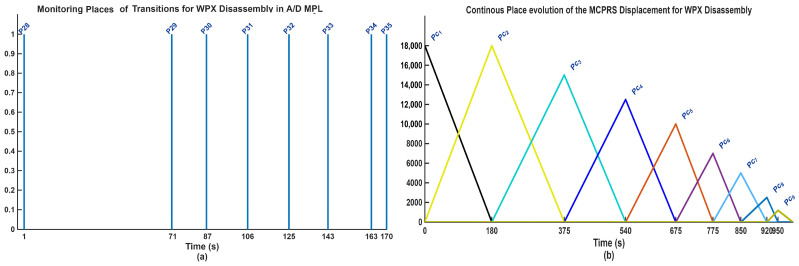
Simulation results of the SHPN_D model for WPX disassembly and component recovery in the A/D/R MPL assisted by MCPRS. (**a**) Evolution of discrete monitoring places showing synchronization signals and event sequencing during disassembly operations. (**b**) Evolution of continuous places $P_{c1}–P_{c9}$ representing the remaining travel distance of the MCPRS along successive trajectory segments for a complete disassembly–recovery cycle.

5. Discrete state of MCPRS disassembly–recovery cycle. A complete operational cycle is performed at constant speed and consists of the following sequence of movements as is shown in Figure 17:
6. Continuous state evolution. Each continuous place corresponds to a segment of the MCPRS trajectory, and its evolution is linear, reflecting constant velocity motion. For visualization efficiency, all continuous states are represented on a single graph (Figure 18b). The most relevant state is $P_{c1}$ (black curve), corresponding to the displacement from the parking position O to position A. Duration is approximately 190 s. MCPRS velocity is 94 mm/s. This duration is consistent with the activation interval of the discrete monitoring place $P_{35}$, confirming correct synchronization between continuous and discrete dynamics.
7. Simulation results and hybrid dynamics of MCPRS. The simulation results obtained using the Sirphyco environment for the SHPN_D model are illustrated in Figure 18a for the monitoring of discrete places and in Figure 18b for the evolution of continuous places. The hybrid modelling approach considers that the position of the MCPRS evolves continuously over time during the disassembly and component recovery processes. The continuous places $P_{c1},\ldots ,P_{c9}$ represent the remaining distance to be travelled by the MCPRS, starting from the parking position O, until the completion of a full disassembly–recovery cycle involving workstations WS2, WS3, and WS4.
8. Hybrid model consistency. The results demonstrate a correct synchronization between Ddiscrete-event dynamics (SHPN_D), governing disassembly operations (ABB IRM), event signalling, task coordination, and continuous dynamics (CPN), governing: MCPRS motion, transport timing, and spatial transitions between workstations.
9. Modelling assumptions. To focus on transport dynamics, the following assumptions were considered:

**Assumption** **M1.** *The manipulation time of the 7-DOF robotic manipulator (Cyton RM) is neglected.*

**Assumption** **M2.** *The fine positioning time provided by the Mobile Visual Servoing System (MVSS) is not included.*

**Assumption** **M3.** *The MCPRS operates at constant velocity.*

10. AI-enhanced perspective. Within the DT framework, the hybrid SHPN–CPN model provides a foundation for AI-driven optimization, enabling predictive estimation of cycle times, adaptive path planning of the MCPRS using reinforcement learning, intelligent synchronization between the A/D/R CPRS and MCPRS, and real-time decision support for disassembly and recovery strategies. These capabilities are particularly relevant in the context of Industry 5.0, where human-centric, flexible, and sustainable manufacturing is emphasized, as well as in Education 4.0/5.0, where such laboratory platforms support advanced learning in cyber–physical systems, AI, and Digital Twins.
11. Formal definition of the SHPN_D model. The disassembly–recovery process is modelled using a SHPN defined as

(10) $$N_{SHPN\text{\_}D}=(P_{d},P_{c},T,Pre,Post,M_{0},\Sigma_{D},{Sync}_{D}).$$

where

(11) $$P_{d}=\left\{P_{1},P_{2},\ldots ,P_{n}\right\}$$

is the finite set of discrete places, representing disassembly states, workstation availability, and monitoring and synchronization conditions;

(12) $$P_{c}=\left\{P_{c1},P_{c2},\ldots ,P_{c9}\right\}$$

is the set of continuous places, representing the remaining travel distances of the MCPRS.

(13) $$T=T_{d}\cup T_{c}$$

is the set of transitions, where

(14) $$T_{d}=\{t_{1},t_{2},\ldots ,t_{m}\}$$

is the discrete transitions (event-driven),

(15) $$T_{c}=\{t_{c1},t_{c2},\ldots ,t_{c9}\}$$

continuous transitions (flow-based).

$Pre,Post:P\times T\to R^{+}$ are the input/output incidence functions.

$M_{0}=\left(M_{d0},M_{c0}\right)$ is the initial marking, with: $M_{d0}\in N^{|P_{d}|}$, and $M_{c0}\in R^{|P_{c}|}$.

$\Sigma_{D}$ is the set of synchronization signals Sync1_d, Sync2_d, Sync3_d, and Sync4_d.

${Sync}_{D}\subseteq T_{d}\times \Sigma$ defines the synchronization mapping between transitions and external signals.

Marking and state evolution. The global marking of the hybrid net is defined as

(16) $$M(t)=(M_{d}\left(t\right),M_{c}\left(t\right)),$$

where $M_{d}(t)$ represents the discrete marking vector (token distribution), and $M_{c}\left(t\right)$ represents the continuous marking vector, corresponding to remaining distances:

(17) $$M_{c}(t)={\left[\begin{array}{cccc} P_{c1}(t) & P_{c2}(t) & \cdots & P_{c9}(t) \end{array}\right]}^{T},$$

The continuous dynamics follow:

(18) $$\frac{dM_{c}(t)}{dt}=C_{c}\cdot v(t),$$

where $C_{c}=Post_{c}-Pre_{c}$ is the continuous incidence matrix, and v(t) is the firing speed vector of continuous transitions.

Assuming constant MCPRS velocity v, each continuous place evolves linearly:

(19) $$P_{ci}(t)=P_{ci}(0)-v\cdot t,$$

until reaching zero, which triggers the corresponding discrete transition.

Discrete–continuous synchronization. The interaction between discrete and continuous dynamics is ensured by event-triggered synchronization.

- When $P_{ci}(t)=0$, a corresponding discrete transition $t_{j}\in T_{d}$ is enabled;
- This transition generates a synchronization signal:

(20) $$Sync_{k}\to t_{j}\to t_{c(i+1)}.$$

Thus, the SHPN ensures correct sequencing of MCPRS movements, synchronization with disassembly operations performed by the A/D/R CPRS, and coordination with storage operations in WS2, WS3, and WS4.

CPN_D for MCPRS motion. The MCPRS displacement is modelled using CPN

(21) $$N_{CPN\text{\_}D}=(P_{c},T_{c},C_{c},M_{c0}),$$

where each place $P_{ci}$ corresponds to a trajectory segment: O→A→B→C→B→D→E→D→F→ O.

The initial marking:

(22) $$M_{c0}={\left[\begin{array}{ccccccccc} d_{OA} & d_{AB} & d_{BC} & d_{CB} & d_{BD} & d_{DE} & d_{ED} & d_{DF} & d_{FO} \end{array}\right]}^{T}$$

represents the segment distances.

12. Interpretation of simulation results. The simulation confirms that the evolution of $P_{c1}$ (Figure 18b, black curve) reaches zero at approximately 190 s, consistent with: MCPRS speed: 94 mm/s, monitoring signal $P_{35}$ (Figure 18a). All continuous places exhibit piecewise linear decreasing behaviour, validating constant-speed motion. Discrete events (Figure 18a) are correctly synchronized with continuous transitions.

**Remark** **3.** *The SHPN_D model thus provides a rigorous and scalable framework for modelling, simulating, and optimizing the disassembly function within the A/D/R MPL, ensuring seamless integration between robotic manipulation, mobile assistance, and intelligent control in a Digital Twin environment.*

Unlike the cylinders and upper assembly components, which are relatively small and must be flexibly transported toward multiple warehouse locations by the MCPRS, the Base component has a different mechanical and logistical role within the A/D/R MPL. The Base acts as the primary support pallet for the workpiece during transportation and assembly operations along the A/D ML conveyor infrastructure. Consequently, its geometry, weight distribution, and standardized positioning are specifically designed to remain compatible with the conveyor-based transport mechanism already integrated into WS1 and the WH1 storage area. For this reason, recovering the Base through the MCPRS would introduce unnecessary mobile manipulation operations and increase transportation complexity without providing significant operational benefit. Instead, after disassembly in the A/D/R CPRS, the Base component is automatically transferred through the conveyor system toward warehouse WH1 in WS1 using the existing pneumatic handling mechanism of the A/D ML. This solution is more efficient because the Base already follows a predefined conveyor-compatible trajectory, no additional robotic grasping and mobile transportation are required, the pneumatic transfer mechanism provides faster and simpler repositioning, and MCPRS resources remain available for more flexible recovery tasks involving distributed components. The pneumatic recycling mechanism of the Base component is fully integrated into the global SHPN synchronization framework. More specifically, the transfer of the Base toward WH1 generates dedicated synchronization events confirming, completion of conveyor transportation, successful pneumatic storage, and warehouse availability.

### 3.9. DT Virtual Layer for the Repair Function

1. Assumptions. The repair process of the defective workpiece WPX within the A/D/R MPL assisted by the two CPRSs is defined under the following assumptions:

**Assumption** **R1.** *All parameters of the repair function (task durations, transport distances, velocities) are known a priori.*

**Assumption** **R2.** *A workpiece *WPX *fails the quality test if it contains heterogeneous cylinders (plastic and metal).*

**Assumption** **R3.** *A defective *WPX *is stored in the right warehouse (WHR) of WS7, and the repair sequence is initiated immediately.*

**Assumption** **R4.** *The defective *WPX *is transported along the A/D ML to the A/D/R CPRS (FC2 position).*

**Assumption** **R5.** *The repair operation consists of replacing the plastic cylinder with a metal one using the ABB IRM.*

**Assumption** **R6.** *The extracted cylinder is recovered by the MCPRS and transported to WS4 (WH4 storage).*

**Assumption** **R7.** *After repair, *WPX *becomes a good product and is transported to WS7 and stored in the left warehouse (WHL).*

**Figure 19:**
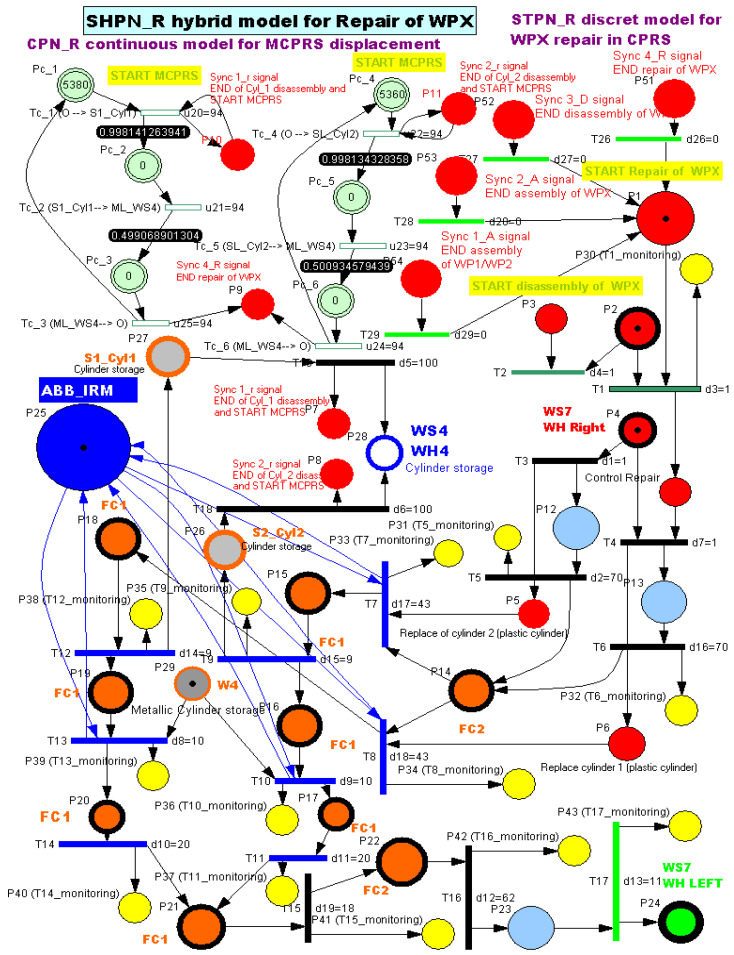
SHPN_R model as VR for repair function of WPX in A/D/R MPL.

2. Task assignment, planning, and synchronization. After the quality test, a defective workpiece WPX is routed to the repair sequence as is shown in Figure 12: The SHPN_R model is shown in Figure 19.

Transport and positioning. WPX is conveyed from WS7 (WHR) to the A/D/R CPRS and positioned at FC1/FC2.

Disassembly and replacement (A/D/R CPRS). The ABB IRM removes the plastic cylinder (position S1 or S2), releases it via the chute, and mounts a metal cylinder from the CPRS warehouse.

Component recovery (MCPRS). The MCPRS detects the released cylinder in S1 or S2, transports it to WS4 (WH4), returns to the parking position (pointO).

Final storage. The repaired WPX is transported form FC2 in CPRS to WS7 in A/D ML and stored in WHL.

3. Synchronization signals. The coordination between discrete (robotic operations) and continuous (MCPRS motion) dynamics is ensured through:
  - Sync1_r: END Cylinder 1 disassembly and START MCPRS,
  - Sync2_r: END Cylinder 2 disassembly and START MCPRS,
  - Sync4_R: END WPX repair and MCPRS in parking position (point O).

4. SHPN_R model, formalism and hybrid dynamics.

The repair process is modelled using SHPN_R, shown in Figure 19, and defined as

(23) $$N_{SHPN\text{\_}R}=\left(P_{d},P_{c},T_{d},T_{c},Pre,Post,M_{0},\Sigma_{R},{Sync}_{R}\right).$$

where

- $P_{d}$: discrete places (repair states, quality control, synchronization),
- $P_{c}=\{P_{c1},P_{c2},P_{c3},P_{c4},P_{c5},P_{c6}\}$: continuous places (MCPRS motion),
- $T_{d}$: discrete transitions (cylinder replacement and storage),
- $T_{c}$: continuous transitions (motion segments),
- $Pre,Post:P\times T\to R^{+}$ are the input/output incidence functions.
- $M_{0}=\left(M_{d0},M_{c0}\right)$ is the initial marking, with: $M_{d0}\in N^{|P_{d}|}$, and $M_{c0}\in R^{|P_{c}|}$;
- $\Sigma_{R}$ is the set of synchronization signals Sync1_r, and Sync2_r;
- ${Sync}_{R}\subseteq T_{d}\times \Sigma$ defines the synchronization mapping between transitions and external signals.

Marking Vector

(24) $$M(t)=(M_{d}(t),M_{c}(t)),$$

with

(25) $$M_{c}(t)={\left[\begin{array}{cccccc} P_{c1}(t) & P_{c2}(t) & P_{c3}(t) & P_{c4}(t) & P_{c5}(t) & P_{c6}(t) \end{array}\right]}^{T}$$

Continuous Dynamics (CPN Representation). The MCPRS motion is modelled by CPN_R:

(26) $$N_{CPN\text{\_}R}=\left(P_{c},T_{c},C_{c},M_{c0}\right),$$

where

(27) $$\frac{dM_{c}(t)}{dt}=C_{c}\cdot v,$$

Assuming constant speed v, the evolution is

(28) $$P_{ci}\left(t\right)=P_{ci}\left(0\right)-v\cdot t.$$

**Figure 20:**
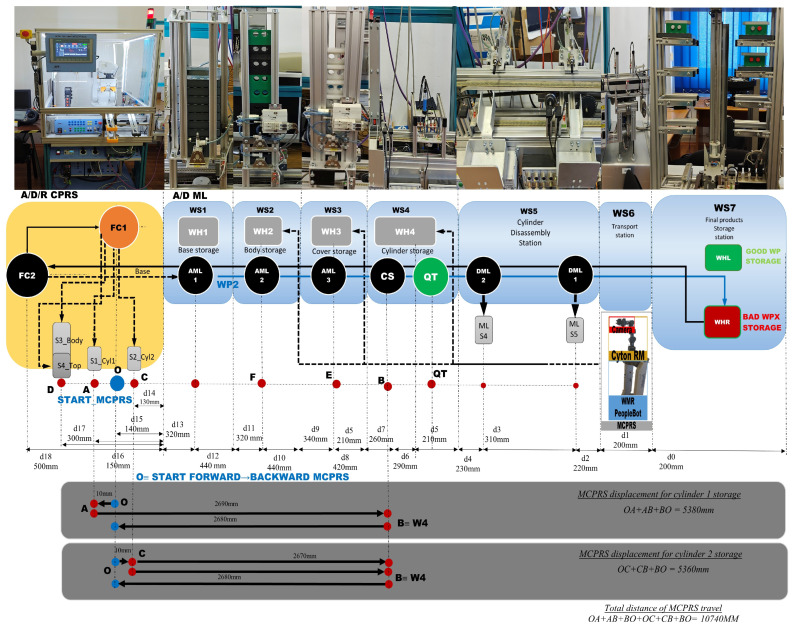
Distances travelled by MCPRS to recover one cylinder during WPX repair.

**Figure 21:**
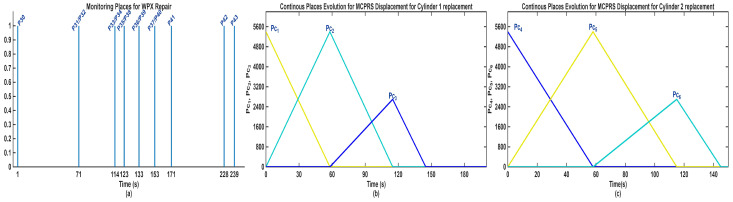
Virtual digital counterpart of the repair function in the A/D/R MPL assisted by MCPRS. (**a**) Monitoring places evolution of the SHPN_R model showing synchronization of repair events and task sequencing. (**b**) Continuous places evolution for MCPRS displacement during Cylinder 1 recovery $\left(O\to A\to B\to O\right)$. (**c**) Continuous places evolution for MCPRS displacement during Cylinder 2 recovery $\left(O\to C\to B\to O\right)$.

Cylinder 1 recovery (Figure 20 and Figure 21b). $P_{c1}\equiv O\to P_{c2}\equiv A$ (distance OA), $P_{c2}\equiv A\to P_{c3}\equiv B$ (distance AB), and $P_{c3}\equiv B\to P_{c1}\equiv O$ (distance BO).

Cylinder 2 recovery (Figure 20 and Figure 21c). $P_{c4}\equiv O\to P_{c5}\equiv C$ (distance OC), $P_{c5}\equiv C\to P_{c6}\equiv B$ (distance CB), $P_{c6}\equiv B\to P_{c4}\equiv O$ (distance BO), as shown in Figure 20.

Hybrid Synchronization Condition. A discrete transition $t_{j}$ is enabled when

(29) $$P_{ci}(t)=0\Rightarrow t_{j}\in T_{d}$$

ensuring synchronization between of cylinder release (discrete event), and MCPRS pickup and transport (continuous evolution).

5. Simulation Results and Interpretation. The simulation results validate the SHPN_R model.

Discrete behaviour (Figure 21a). Monitoring places confirm correct sequencing of quality test, cylinder disassembly, replacement (repair) and storage. Continuous behaviour (Figure 21b,c). Linear decrease of $P_{ci}$ confirms constant MCPRS velocity, Cylinder 2 trajectory is slightly shorter than Cylinder 1, and synchronization signals correctly trigger motion phases.

**Remark** **4.** *The SHPN_R model thus provides a rigorous and scalable framework for modelling, simulating, and optimizing the repair function within the A/D/R MPL, ensuring seamless integration between robotic manipulation, mobile assistance, and intelligent control in a Digital Twin environment.*

In the proposed experimental A/D/R MPL, the quality classification of WPX workpieces is exclusively based on the material combination of the two cylinders detected during the quality inspection stage in WS7. According to the experimental production rules adopted in the platform, for the current experimental implementation, this rule-based material verification is considered sufficient to classify the repaired WPX as a compliant product. Therefore, an additional complete quality inspection stage is not physically repeated after repair. Nevertheless, the repair sequence still includes implicit verification mechanisms through PLC sensor validation, synchronization signals, robotic manipulation confirmation, and MVSS visual positioning supervision. These mechanisms ensure that the replacement operation has been successfully completed, the correct metallic cylinder has been inserted, and the repaired WPX is properly transferred toward the compliant-product warehouse (WHL). the current qualification strategy is based on the experimental rule adopted for the laboratory platform, where compliance is determined solely by the final cylinder material configuration. The manuscript also clarifies that in real industrial applications, additional post-repair quality inspection procedures could be integrated, including, visual inspection, dimensional verification, force/torque validation, sensor-based testing, or AI-assisted quality assessment. Such extensions would allow the repaired workpiece to undergo a complete reinspection cycle before being classified as compliant in large-scale industrial deployment scenarios.

### 3.10. DT Virtual Layer of the MCPRS

**Figure 22:**
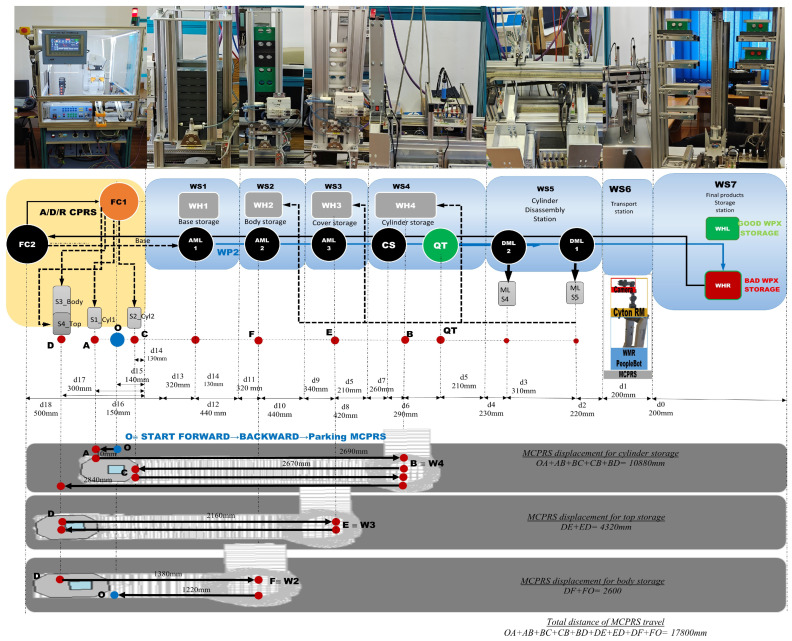
Virtual Digital Twin of the MCPRS in the MPL environment with layout of the MPL including workstations (WS1–WS7), storage locations (WH1–WH7), CPRS interface, MCPRS trajectories, in MobileSim, for disassembly and repair operations, including pickup points (S1–S4) and parking position O, and forward/backward motion paths with associated travel distances used for trajectory optimization.

The DT of the MCPRS is implemented using MobileSim to simulate, validate, and optimize the navigation and task execution of the MCPRS within the Mechatronic Production Line (MPL). A detailed virtual model of the MPL, including all workstations (WS1–WS7), storage locations (WH1–WH7), slides, and transportation paths, is constructed to reproduce the real system layout, as shown in Figure 22. This virtual environment enables the MCPRS counterpart to perceive spatial constraints, predefined trajectories, and potential obstacles, allowing realistic and collision-free path planning. The simulation ensures that trajectory generation satisfies operational constraints while minimizing travel time.

1. Trajectory modelling and control. The MCPRS motion is modelled using the Discrete-Time, Terminal-Twisting trajectory Sliding Mode Control (DT-TTSMC) strategy. This approach ensures robustness against disturbances and modelling uncertainties by continuously correcting trajectory deviations. The DT-TTSMC controller reproduces the kinematic and dynamic behaviour of the PeopleBot wheeled mobile robot, including acceleration, deceleration, forward/backward motion, and turning maneuvers. Consequently, realistic motion profiles are obtained for navigation in constrained industrial environments.
2. Synchronization with MPL operations. The MCPRS operation is synchronized with the MPL processes through CPNs, ensuring coherence with the global control architecture presented in Figure 7. Two models are considered:
  - $CPN_{D}$: synchronization during disassembly,
  - $CPN_{R}$: synchronization during repair.

The synchronization mechanism is expressed as

(30) $$t_{i}\overset{Sinck_{d}/r}{\to }t_{c},$$

which establishes the coupling: Discrete events (CPRS)⇒Continuous MCPRS motion.

The triggering signals are

- $\left\{Sync1\text{\_}d,Sync2\text{\_}d,Sync3\text{\_}d,Syn4\text{\_}d\right\}$ for disassembly;
- $\left\{Sync1\text{\_}r,Sync2\text{\_}r\right\}$ for repair.

3. MCPRS operation during disassembly. The MCPRS starts from the parking position O, located in front of the CPRS slides. It sequentially collects disassembled components from the slides and transports them to the corresponding warehouses (Figure 22).

Cylinder transport to WS4–WH4:

- O→A: pickup of Cylinder 1 (Slide S1_Cyl1),
- A→B: delivery to WH4,
- B→C: movement toward Cylinder 2 (Slide S2_Cyl2),
- C→B: delivery to WH4.

Top component transport to WS3–WH3:

- B→D: movement toward Top (Slide S4_Top),
- D→E: delivery to WH3.

Body component transport WS2–WH2:

- E→D: return toward CPRS,
- D→F: pickup of Body (Slide S3_Body),
- F→WH2: delivery to WH2.

All trajectories are executed at constant velocity. After each delivery, the MCPRS returns along the same path to continue the sequence or return to the parking position.

4. MCPRS Operation During Repair. During repair, only one cylinder is replaced at a time. Therefore, the MCPRS follows simplified trajectories:
  - O→A→B: Cylinder 1 transport to WH4,
  - O→C→B: Cylinder 2 transport to WH4.

After completing the task, the MCPRS returns to the parking position O.

5. End-effector control and visual servoing. Accurate pick-and-place operations are ensured by the integration of a 7-DOF RM and a MVSS. The visual feedback enables precise alignment during pickup and placement at both the CPRS slides and the A/D ML warehouses.
6. Software Implementation. The DT-TTSMC strategy is implemented in Visual C++ (MSVC v143, Visual Studio 2022) while ARIA (Advanced Robotic Interface for Applications) libraries are used for MCPRS control. In the absence of the physical MCPRS, MobileSim automatically activates its virtual counterpart, ensuring uninterrupted simulation and testing (Figure 22).

**Remark** **5.** *The virtual MCPRS provides a reliable platform for validation of forward and backward trajectories, optimization of travel distances and execution time, synchronization with MPL processes via* $CPN_{D}$ *and* $CPN_{R}$ *, integration into the overall DT framework.*

## 4. DT Physical Layer Control of the A/D/R MPL Assisted by Dual CPRS

### 4.1. Communication and Control Architecture of the A/D/R MPL

The real-time control architecture of the A/D/R MPL assisted by dual CPRS—namely:

- the A/D/R-CPRS (with ABB IRM),
- the mobile CPRS (MCPRS),

is implemented through a hierarchical, distributed, and networked control structure, presented in Figure 5, Figure 6 and Figure 7. The system integrates SCADA, HMI, PLCs, and industrial communication protocols to ensure real-time monitoring, supervision, and control [51,52,53,54,55,56].

Core functionalities. The SCADA system, combined with local and remote HMIs, enables:

- Data acquisition by real-time acquisition of digital and analogue signals from A/D/R-CPRS, A/D ML (7 workstations), and MCPRS for full system observability.
- Bidirectional communication between subsystems via master PLC, Siemens S7-1200, slave PLCs, Siemens S7-300 (WS1–WS7), and interface module: CM 1242-5 (Profibus DP compliant, IEC 61158).
- CM 1242-5 module manages cyclic data exchange, ensures deterministic communication, and offloads communication tasks from the master PLC.
- Human–machine interaction (HMI). Interfaces (KTP700, TP177, Remote PC) provide intuitive GUIs aligned with Industry 5.0 human-centric design, improv usability, supervision, and decision-making.
- Remote supervision and control. SCADA enables remote operation via networked infrastructure, allowing synchronization of signal monitoring, fast intervention, and centralized control.

Industrial Communication and Digital Integration. The architecture supports a multi-protocol industrial network by

- PROFIBUS DP → PLC-to-PLC communication (A/D ML),
- PROFINET/Ethernet → high-speed local communication,
- MODBUS TCP → MCPRS–PLC synchronization,
- OPC UA → interoperability, cloud integration, IoT connectivity.

**Remark** **6.** *The use of OPC UA ensures platform independence, scalability, and integration with DT environments.*

### 4.2. AI and Industry/Education 4.0/5.0 Integration

The A/D/R MPL assisted by dual CPRS is laboratory-scale system, and the platform aligns with:

- Industry 4.0 by connectivity, automation, DT,
- Industry 5.0 by human-centric control, adaptability,
- Education 4.0/5.0 by experiential learning, remote labs.

AI is integrated through:

- Vision-based control (MVSS using image processing),
- Potential extensions (learning-based optimization, predictive control).
- Intelligent supervision via SCADA analytics.

### 4.3. Real-Time Control and Synchronization of MCPRS Subsystems

**Figure 23:**
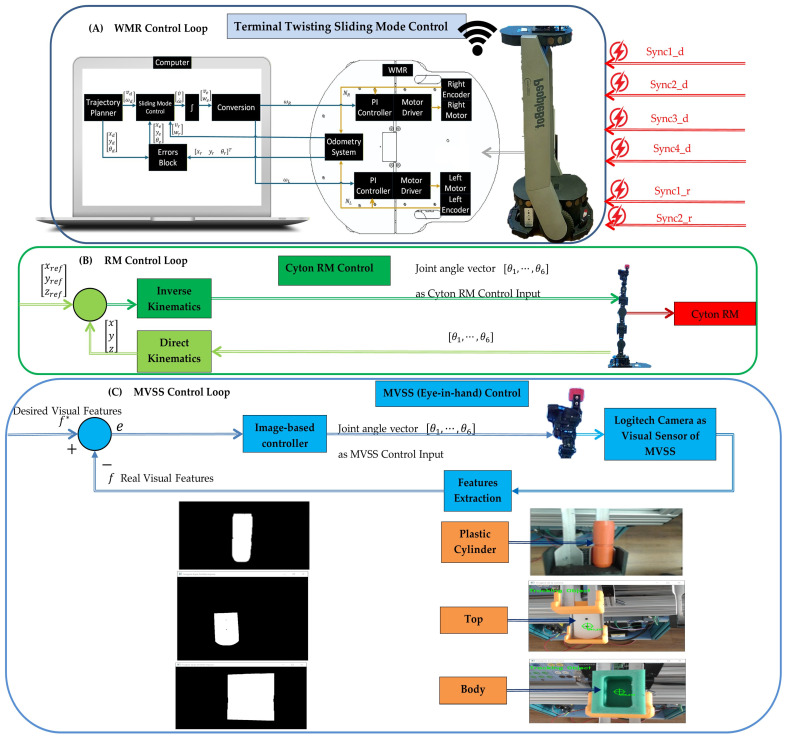
MCPRS’s control loops for the triplet, PeopleBot WMR, Cyton 1500 RM, and MVSS.

The MCPRS is a mobile cyber–physical robotic system composed of PeopleBot WMR (2DW/1FW mobile robot), Cyton 1500 RM (7-DOF manipulator), and MVSS (eye-in-hand visual servoing system with camera). These subsystems operate through three coordinated control loops all synchronized via the Remote PC (SCADA server), as presented in Figure 23.

1. WMR control Loop ensures mobility control between slides of CPRS and WHs storage location of A/DML, using DT-TTSMC as control method, odometry and encoder date as feedback, and Wi-Fi (TCP/IP and ARIA library) as communication. This loop ensures robust trajectory tracking under uncertainties.
2. Cyton 1500 RM control loop (Manipulation & Synchronization) ensures execution of manipulation tasks (pick/place, recovery) and uses inverse kinematics (IKC) for positioning and Modbus TCP for synchronization with master PLC as control method. It communicates by TCP/IP with Remote PC. This loop ensures precise coordination with A/D/R MPL processes.
3. MVSS control loop (vision-based precision control) ensures fine positioning of the end-effector and uses image-based visual servoing (IBVS) by image moments, and feature extraction (OpenCV, V: 4.13.0, MATLAB, V: R2025b)). Its function is real-time correction of positioning errors. This loop introduces AI-driven perception and adaptive control.

The MVSS integrated into the MCPRS is responsible for high-precision visual localization and final alignment during assembly, disassembly, and repair operations. The synchronization mechanism between the MVSS, the PLC layer, and the physical execution phase requires further clarification. In the proposed architecture, the MVSS does not operate independently; instead, it is tightly synchronized with the Siemens S7-1200 PLC, the Node-RED orchestration layer, and the SHPN coordination framework through event-triggered synchronization signals and OPC-UA communication. The synchronization logic between visual perception and physical execution is organized as a closed-loop cyber–physical coordination mechanism. The PLC first supervises the coarse positioning phase of the MCPRS using the discrete-event SHPN model and the TTSMC navigation controller of the PeopleBot WMR. Once the MCPRS reaches a predefined target region near the workstation or warehouse, the master PLC generates a visual-servo activation event which activates the MVSS subsystem and switches the control mode from coarse navigation to fine visual positioning. At this stage, the Logitech high-definition camera mounted on the Cyton 1500 manipulator continuously acquires images of the target component, markers, or workstation reference points. The IBVS algorithm extracts visual features such as centroids, contours, fiducial markers or object poses and computes the image tracking error. The visual controller then generates corrective commands for the Cyton 1500 manipulator and, if necessary, micro-positioning commands for the PeopleBot WMR. During this phase, the PLC temporarily inhibits the execution of grasping or assembly actions until the visual error satisfies the admissibility condition. When this condition is satisfied, the MVSS generates the synchronization signal which confirms successful visual alignment and precise positioning. This signal is transmitted through OPC-UA to the PLC and Node-RED supervision layer.

- Inverse Kinematic Control (IKC). Let the desired end-effector position be

(31) $$x_{d}=[x_{d},y_{d},z_{d}{]}^{T},$$

and the joint vector:

(32) $$q=[\theta_{1},\theta_{2},\ldots ,\theta_{6}{]}^{T}.$$

The forward kinematics is

(33) x=f(q).

The inverse kinematics computes

(34) $$q_{d}=f^{-1}(x_{d}).$$

For differential motion:

(35) $$\dot{x}=J(q)\dot{q}.$$

the joint velocity control is

(36) $$\dot{q}=J^{†}(q)\;{\dot{x}}_{d},$$

where $J^{†}$ is the pseudoinverse of the Jacobian.

- MVSS (Image-Based Control—IBVS). s is current visual features (centroid), $s^{*}$ is desired visual features.

The image error is

(37) $$e=s-s^{*},$$

and interaction matrix (image Jacobian) is $L_{s}$.

The control law is

(38) $$v=-\lambda L_{s}^{†}e,$$

where v is camera/end-effector velocity, and λ>0 is control gain.

- Tracking error model (Cartesian space). The position error is defined as

(39) $$e_{x}=x_{d}-x,$$

with the components

(40) $$e_{x}=x_{d}-x,e_{y}=y_{d}-y,e_{z}=z_{d}-z.$$

The control objective is

(41) $$\underset{t\to \infty }{lim}e_{x}(t)=0.$$

- Closed-loop combined control (IKC + MVSS). The hybrid control law can be expressed as

(42) $$\dot{q}=J^{†}\left({\dot{x}}_{d}-\lambda L_{s}^{†}(s-s^{*})\right).$$

The Equation (41) combines model-based control (IKC) and sensor-based correction (vision feedback). Follows an integration of IKC with Visual Servoing and TTSMC (for WMR).

- Task-space error definition. Define the global task-space error:

(43) $$e=\left[\begin{array}{c} e_{x}\\ e_{s} \end{array}\right]=\left[\begin{array}{c} x_{d}-x\\ s-s^{*} \end{array}\right],$$

where $e_{x}$ is the cartesian error (RM + WMR contribution), and $e_{s}$ is thevisual feature error.

- Kinematic coupling (WMR + RM). The end-effector velocity depends on both manipulator joints q, mobile base configuration $p=[x_{m},y_{m},\theta {]}^{T}$ and

(44) $$\dot{x}=J_{q}(q)\dot{q}+J_{p}(p)\dot{p}.$$

- Combined control law. Manipulator control (IKC + Vision)

(45) $$\dot{q}=J_{q}^{†}\left({\dot{x}}_{d}-J_{p}\dot{p}-\lambda L_{s}^{†}e_{s}\right),$$

and WMR Control via TTSMC by defining the tracking error for mobile platform (PeopleBot WMR):

(46) $$e_{p}=p_{d}-p,$$

where mobile WMR configuration $p=[x_{m},y_{m},\theta {]}^{T}$, and p_d_ is the desired WMR configuration.

- Terminal sliding surface (TTSMC). Define nonlinear sliding surface:

(47) $$s_{p}={\dot{e}}_{p}+\alpha e_{p}+\beta |e_{p}{|}^{\gamma }sign(e_{p}),$$

where 0<γ<1(terminal convergence), α,β>0.

- TTSMC law. The control input for PeopleBot WMR is

(48) $$u=u_{eq}-K\;sat\left(\frac{s_{p}}{\phi }\right)$$

where $u_{eq}$ is the equivalent control (model-based), K>0 is the gain, and ϕ is the boundary layer thickness.

- $H_{\infty }$ robustness formulation for the triplet PeopleBot WMR, Cyton RM, and MVSS consists of defining the augmented error vector with the errors from (40), (46), and (48):

(49) $$z=\left[\begin{array}{c} e_{x}\\ e_{s}\\ s_{p} \end{array}\right],$$

and disturbances input (exogenous), $w\left(t\right)$, modelling errors (IKC), vision noise, external perturbations. In state-space representation the closed-loop system can be abstracted as

(50) $$\dot{z}=Az+Bw.$$

Define performance output (quality):

(51) y=Cz,

where y represents tracking errors and sliding variables.

The $H_{\infty }$ objective consists of designing the control input so that the transfer from exogenous to quality should be as small as possible:

(52) $$\int_{0}^{\infty }{∥y(t)∥}^{2}dt\leq \gamma^{2}\int_{0}^{\infty }{∥w(t)∥}^{2}dt,$$

where γ>0 is the disturbance attenuation level.

Lyapunov-based $H_{\infty }$ condition consists of choosing a quadratic Lyapunov function:

(53) $$V=z^{T}Pz,P>0,$$

and the condition for $H_{\infty }$ performance is

(54) $$\dot{V}+y^{T}y-\gamma^{2}w^{T}w\leq 0,$$

that leads to the standard LMI condition (design form)

(55) $$\left[\begin{array}{cc} A^{T}P+PA+C^{T}C & PB\\ B^{T}P & -\gamma^{2}I \end{array}\right]<0.$$

It can conclude that

- DT-TTSMC ensures robustness in PeopleBot WMR against matched disturbances;
- IBVS ensures exponential decay of visual errors generated by MVSS with high-definition Logitech camera,
- IKC provides nominal trajectory tracking of Cyton 1500 RM,
- Disturbance energy is attenuated below γ,
- Closed-loop system is robust to modelling errors, sensor noise, and external disturbances.

The proposed control framework guarantees finite-time convergence of the mobile base via TTSMC, while ensuring $H_{\infty }$ robustness of the overall MCPRS-assisted manipulation system against bounded disturbances and modelling uncertainties. In a future paper, we will explicitly address finite-time convergence proof for TTSMC and an $H_{\infty }$ robustness formulation tailored for the triplet WMR–RM–MVSS. The stability is rigorously proven using Lyapunov theory and LMI-based performance conditions. Remote PC (SCADA server) synchronizes all control loops, manages communication between MCPRS, A/D/R CPRS, A/D ML, and integrates physical and computational layers. The parameters used in the proposed control architecture, including the DT-TTSMC, IBVS controller, the $H_{\infty }$ robust controller, and the fractional-order compensation terms, were not selected empirically or arbitrarily. Their tuning process was carried out according to stability conditions, physical constraints of the MCPRS, sampling limitations, actuator capabilities, and experimental performance requirements of the A/D/R MPL assisted by the dual CPRS. The parameter selection strategy combines theoretical stability analysis, Lyapunov-based convergence conditions, robustness requirements, and experimental fine-tuning performed on the real cyber–physical platform. For the DT-TTSMC controller applied to the PeopleBot WMR navigation, the sliding surface parameters were selected to ensure finite-time convergence while maintaining robustness against model uncertainties, wheel slip, payload variations, and communication delays. The switching gains of the twisting controller were determined according to the upper bound of external disturbances and modelling uncertainties. The sampling time of the DT-TTSMC controller was selected considering the PLC scan cycle, ROS communication delays, OPC-UA transmission latency, and the dynamic response bandwidth of the PeopleBot WMR. For the IBVS controller integrated within the MVSS, the gain matrix was selected based on the interaction matrix (image Jacobian) and the desired convergence rate of visual features in the image plane.

The proposed DT architecture establishes a bidirectional synchronization mechanism between the physical MPL, the CPRS and MCPRS, and the virtual SHPN-based simulation environment. To guarantee reliable cyber–physical interaction and real-time supervisory control, the synchronization performance between the real and virtual layers was carefully evaluated in terms of communication delay, event synchronization latency, data refresh period, and state consistency. The virtual–real synchronization mechanism is implemented through a multilayer communication architecture integrating Siemens S7-1200 PLCs, OPC-UA industrial communication, Node-RED orchestration, ROS middleware, MQTT/IoT–Cloud services, and the SHPN-based Digital Twin simulation engine. The synchronization process operates according to a cyclic update mechanism in which the physical process states, sensor measurements, robotic positions, SHPN markings, and event transitions are continuously transmitted from the real system toward the DT, while control decisions, optimized trajectories, and supervisory commands are transmitted back to the physical layer.

In the proposed SHPN-based supervisory architecture, synchronization signals are not only used to trigger transitions under normal conditions, but also to supervise the consistency and safety of the overall cyber–physical process. Each synchronization signal is associated with timing constraints, expected transition sequences, watchdog monitoring conditions, and supervisory validation rules implemented through the PLC, Node-RED orchestration layer, and SHPN synchronization places. When a synchronization signal is not triggered within the expected temporal interval, the corresponding SHPN transition remains disabled, and the system automatically enters a controlled waiting state. During this phase the current process marking is preserved, downstream transitions are inhibited, and the PLC supervises timeout conditions through dedicated monitoring timers. If the delay exceeds a predefined threshold, an alarm state is generated and transmitted to the SCADA system, Node-RED dashboard, and the HMI supervisory interface. This alarm mechanism allows operators to identify workstation blocking, MCPRS navigation delays, failed visual servoing operations, communication interruptions, or incomplete robotic manipulation tasks. In the case of abnormal synchronization ordering, the SHPN structure inherently prevents illegal transition firing because each transition is conditionally enabled only if the required synchronization places contain valid tokens. Consequently, out-of-sequence synchronization signals cannot activate unsafe operations. If inconsistent synchronization conditions persist, the system enters a safety stop mode in which mobile robot motion is suspended, manipulator commands are inhibited, conveyor operations are stopped, and the system waits for operator validation or supervisory recovery procedures. Additionally, manual intervention can be enabled through the SCADA/HMI, allowing operators to reset synchronization states, restart blocked transitions, validate completed operations, or switch the system into manual operation mode. The proposed synchronization architecture therefore supports four supervisory response modes under abnormal synchronization conditions: waiting mode for temporary delays, alarm mode for timeout detection, safety stop mode for inconsistent or unsafe synchronization states, and manual intervention mode for operator-assisted recovery.

These mechanisms significantly improve the robustness and reliability of the SHPN-based cyber–physical coordination framework and ensure safe operation of the IoT–Cloud-based A/D/R MPL assisted by dual CPRS under non-ideal industrial operating conditions.

### 4.4. Real-Time Validation and AI-Based Visual Tracking in A/D/R MPL Assisted by MCPRS

**Figure 24:**
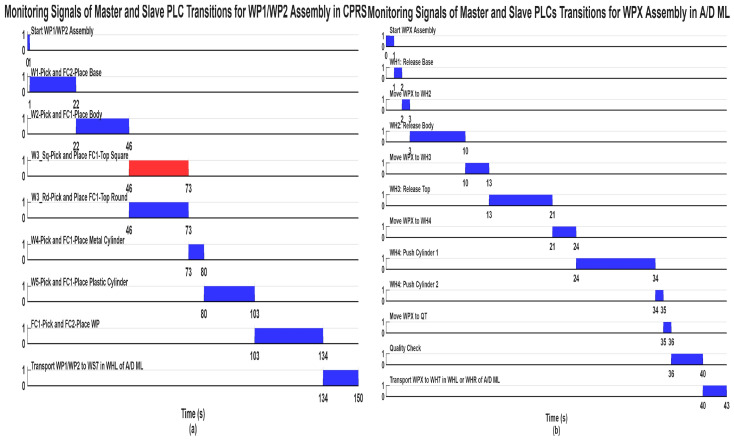
Monitoring signals of master and slave PLC transition for the assembly process in the A/D/R MPL. (**a**) Monitoring signals for the assembly of WP1/WP2 in the A/D/R CPRS. (**b**) Monitoring signals for the assembly of WPX in the A/D ML.

**Figure 25:**
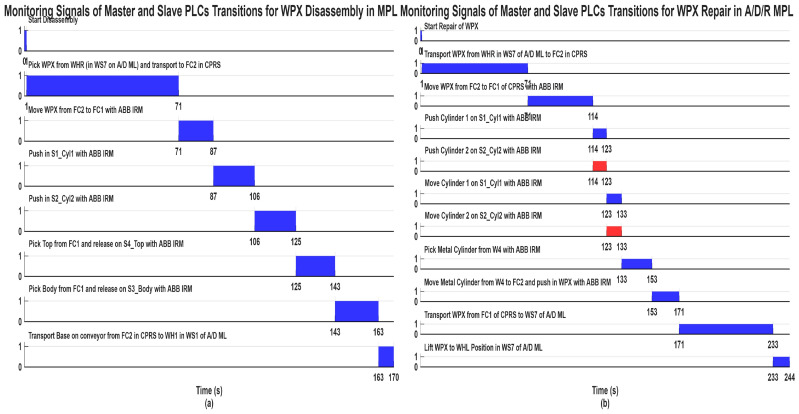
Monitoring signals of master and slave PLC transition for disassembly process in the A/D/R MPL. (**a**) Monitoring signals for the assembly of WP1/WP2 in the A/D/R CPRS. (**b**) Monitoring signals for the assembly of WPX in the A/D ML.

The developed STPN_A, SHPN_D, SHPN_R, CPN_D, and CPN_R models, corresponding respectively to assembly, disassembly, and repair processes, are transposed into a real-time control application through their implementation within the Siemens SCADA platform. This implementation is achieved by interfacing the models with synchronized signals acquired from the real process via PLCs and distributed sensors. Following deployment, the real-time behaviour of the A/D/R MPL assisted by MCPRS is experimentally validated using the monitoring signals of master and slave PLC transitions illustrated in Figure 24 and Figure 25. These results are compared with those obtained from Sirphyco-based simulation, which models the hybrid dynamics (discrete–continuous states) of the corresponding SHPN frameworks, presented in Figure 15, Figure 18 and Figure 21.

1. Real-Time Synchronization and Model Validation. The timing signals used in the real system play a critical role in validating transitions within the STPN_A, SHPN_D, and SHPN_R models. Each transition is conditionally enabled by component release signals from the CPRS, component recovery and storage signals executed by the MCPRS within A/D ML warehouses (WH1–WH7). This synchronization mechanism ensures consistency between simulated and real system behaviour, correct sequencing of assembly, disassembly, and repair operations, coordinated activation of MCPRS actions. Furthermore, synchronization signals initiate robot activation (WMR + RM), task execution across workstations, and real-time monitoring and supervisory control via SCADA.
2. Real-Time Control Performance. The control of the PeopleBot WMR within the MCPRS is achieved using a Discrete-Time Terminal Sliding Mode Control (DT-TTSMC) strategy based on the kinematic model. This approach provides robust trajectory tracking, fast convergence, resilience to uncertainties and disturbances. Through the coordinated control of both MCPRS and A/D/R MPL, the system achieves optimized cycle times for assembly, disassembly, and repair processes, efficient logistics between CPRS and warehouse stations, and improved global system productivity.
3. AI-Based Visual Detection and Tracking (MVSS). The MVSS integrates AI-based image processing for real-time object detection and tracking. The processing pipeline consists of four main stages:
  - Stage 1: Image acquisition and colour space transformation. Images are captured using a Logitech HD camera in BGR format (default in OpenCV). Each frame, with a resolution of 640 × 480 pixels, is converted into the HSV (Hue–Saturation–Value) colour space. The HSV representation is preferred because separates luminance (Value) from chromatic information (Hue, Saturation), provides robustness against illumination variations and improves detection reliability in dynamic environments.
  - Stage 2: Colour filtering and segmentation. A colour-based thresholding process is applied using predefined HSV bounds lower bound and upper bound define the target colour range, and a binary mask is generated to isolate relevant pixels. The minimum area threshold removes noise and small artefacts, and the maximum area threshold avoids false detections from large objects. This stage performs implicit morphological filtering and reduces computational complexity.
  - Stage 3: Centroid detection and localization. For each detected object: the centroid (geometric centre) is computed using pixel distribution. The centroid is marked (⊕) and used as a compact spatial representation. This enables efficient communication with control modules, and real-time feedback for robotic positioning.
  - Stage 4: Morphological Processing and Contour Extraction. To refine detection erosion removes residual noise and dilation restores object integrity. This sequence (erosion → dilation) forms an opening operation, widely used in image processing. Additionally, contour detection (by algorithms such as Canny or find Contours from OpenCV) extracts object boundaries with high precision.

### 4.5. Experimental Results and Interpretation

**Figure 26:**
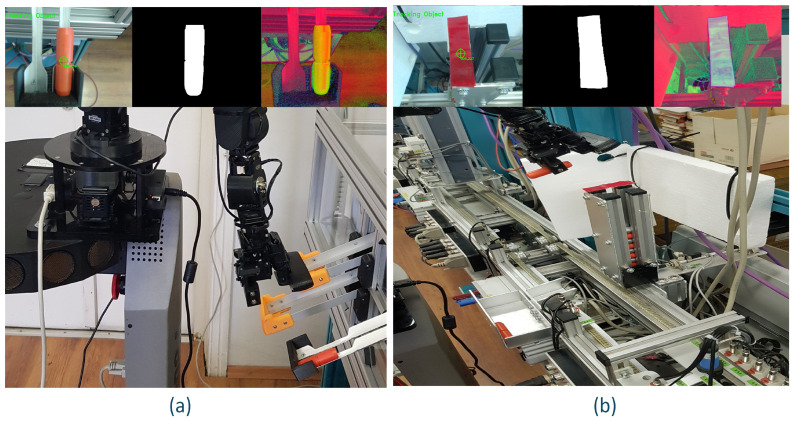
Vision-based detection and manipulation of plastic cylinder using MCPRS. (**a**) Picking operation from the S1_cyl station in CPRS: real-time object detection using the MVSS, including HSV-based segmentation, centroid extraction, and visual tracking of the plastic cylinder. (**b**) Placing operation in warehouse WH4 (WS4 of A/D ML): visual alignment and positioning of the end-effector based on real-time feedback from the MVSS for accurate component placement.

**Figure 27:**
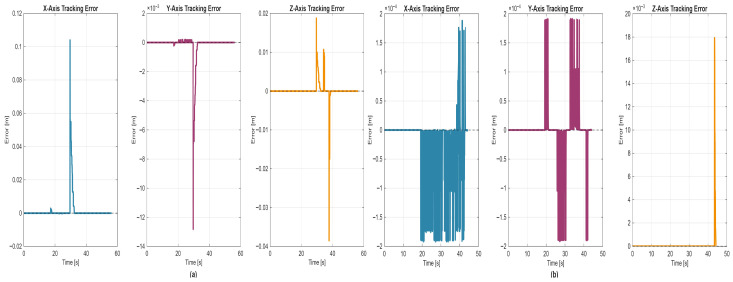
End-effector Cartesian tracking errors of the Cyton 1500 RM during manipulation tasks. (**a**) X-, Y-, and Z-axis tracking errors during the picking operation from S1_cyl, showing transient deviations and convergence behaviour under visual servoing. (**b**) X-, Y-, and Z-axis tracking errors during the placing operation in WH4, illustrating improved stability and precision due to closed-loop feedback control.

**Figure 28:**
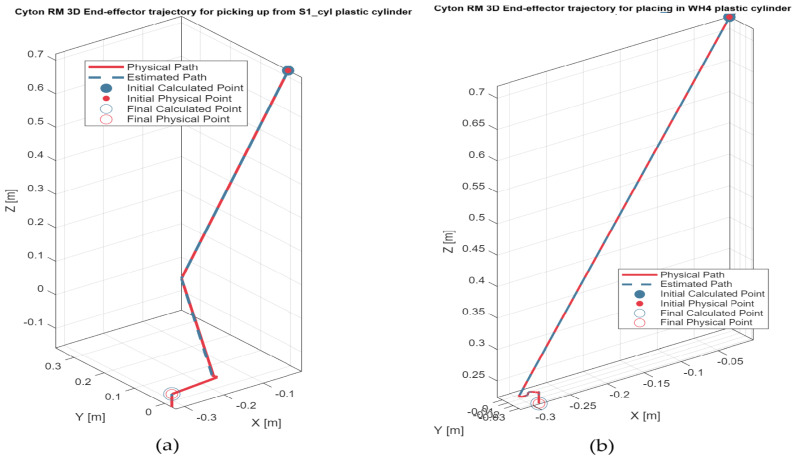
3D end-effector trajectories of Cyton 1500 RM for pick-and-place operations. (**a**) Picking trajectory from S1_cyl1: comparison between the estimated trajectory (inverse kinematics-based) and the physical trajectory measured in real time, including initial and final positions. (**b**) Placing trajectory in WH4: correspondence between planned and executed motion, demonstrating high accuracy and synchronization of the control system.

**Figure 29:**
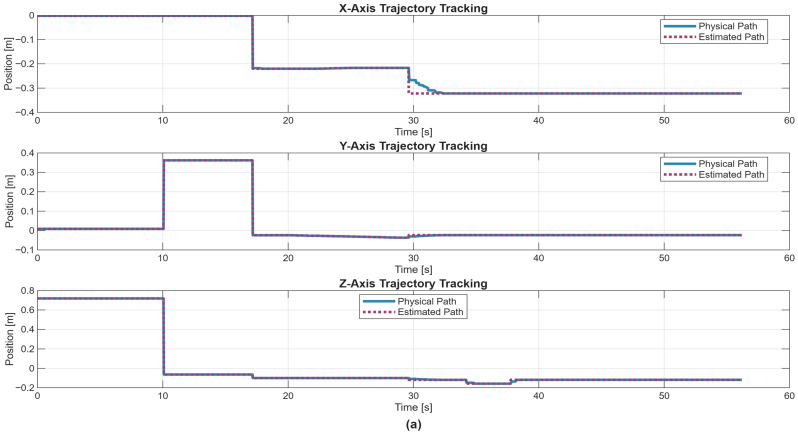

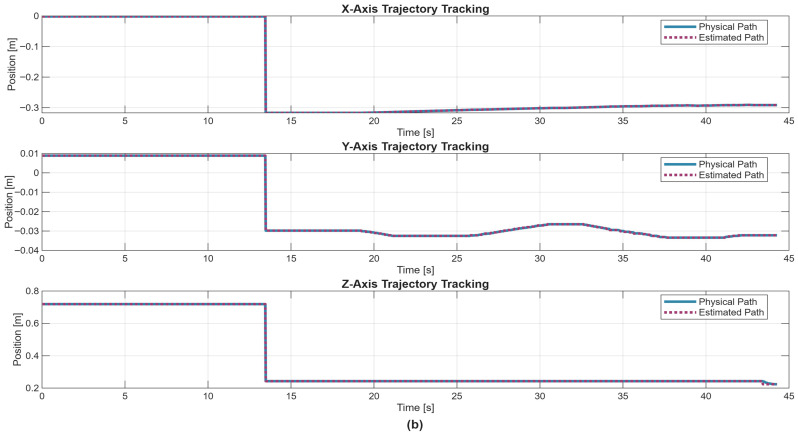
Time evolution of the X–Y–Z end-effector position during real-time MCPRS-assisted A/D/R MPL operations: (**a**) Position tracking during the picking phase from S1_cyl1 in CPRS. (**b**) Position tracking during the placing phase into warehouse WH4 of the A/D ML.

- Vision-based detection and picking operation. During the picking operation from the S1_cyl1 station of the CPRS, the MVSS performs real-time detection of the plastic cylinder. The upper-left region of Figure 26a shows: object detection using HSV-based segmentation, and centroid localization of the cylinder. The extracted visual features are used to compute positioning errors, generate corrective commands for the RM end-effector. This process enables accurate grasping despite uncertainties in object position. The visual results from upper medallion demonstrate real-time tracking with centroid overlay and positional data (left), HSV-based processed representation (middle), and binary mask after segmentation (right). This pipeline ensures robust detection under varying lighting conditions, accurate real-time tracking, and reliable integration with MVSS control loop.
- Vision-based placement operation. For the placing operation in warehouse WH4 (WS4 of A/D ML), The MVSS detects the target placement location, as illustrated in Figure 26b. The system performs: visual alignment, trajectory correction, and final placement using feedback control. This ensures precise deposition of the component into the designated storage location.
- End-effector tracking performance. The tracking performance of the Cyton RM is evaluated using Cartesian position errors (X, Y, Z): Figure 27a—tracking errors during picking, and Figure 27b—tracking errors during placing operation The results indicate small steady-state errors, transient deviations during motion phases, and rapid convergence due to closed-loop visual feedback. This confirms the effectiveness of the vision-based servoing combined with IKC.
- 3D end-effector trajectory analysis. The spatial trajectories of the end-effector are illustrated in Figure 28a—3D trajectory for picking, and in Figure 28b—3D trajectory for placing. Each figure compares estimated trajectory (computed via IKC), physical trajectory (measured in real time), and initial and final positions (calculated vs. actual). The close overlap between estimated and real trajectories demonstrates high modelling accuracy, effective synchronization between control and execution, and robustness of the overall control architecture.
- Time evolution of the X–Y–Z end-effector position during real-time MCPRS-assisted A/D/R MPL operations is presented in Figure 29a—Position tracking during the picking phase from S1_cyl1 in the CPRS, and in Figure 29b—position tracking during the placing phase into warehouse WH4 of the A/D ML.

The observed transient deviations (notably around switching instants at t≈30 s for picking and t≈14 s for placing) correspond to discrete-event transitions in the synchronized SHPN model and to changes between motion primitives (approach, grasp, transport, and release phases). These transitions are governed by PLC-triggered events and validated through real-time sensor feedback. Each subplot presents the comparison between the physical trajectory and the estimated trajectory obtained via the hybrid control scheme. The close overlap between the two signals confirms high tracking accuracy and robustness of the proposed control architecture.

**Figure 30:**
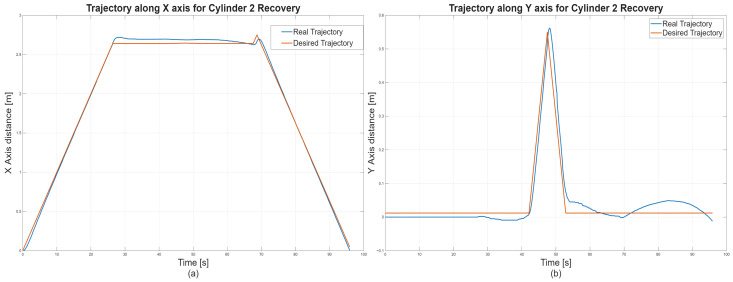
Real-time tracking performance of the MCPRS, specifically the PeopleBot WMR, (**a**) along X-axis and (**b**) along Y-axis, during the recovery and transport of Cylinder 2 from the CPRS station (S2_Cyl2) to warehouse WH4 in WS4 of the A/D ML, followed by return to the parking position.

Figure 30a illustrates the trajectory along the longitudinal (X) axis, with the proposed discrete-time terminal twisting sliding mode controller (DT-TTSMC). It ensures accurate tracking of the desired trajectory with smooth transition at trajectory switching points (~70 s), indicating robustness to directional changes A small overshoot is observed at the beginning of the constant-position phase, which is rapidly attenuated due to the nonlinear terminal sliding surface. The H∞ performance bound, ensuring that the tracking error satisfies

(56) $${∥e(t)∥}_{2}\leq \gamma {∥w(t)∥}_{2}$$

where γ=0.05 is obtained via LMI-based synthesis. The real trajectory remains entirely within this bound, validating robustness against disturbances and modelling uncertainties. Figure 30b shows the lateral (Y-axis) trajectory, which reflects fine positioning and alignment at the target workstation with low steady-state deviation during straight motion, indicating effective disturbance rejection, sharp transient peak (~45–50 s) corresponding to the alignment phase at WH4, rapid error attenuation after the peak, confirming finite-time convergence and small oscillations during the return phase due to coupling effects and discrete-time implementation Despite these challenges, the tracking error remains bounded and within the predefined H∞ robustness envelope, demonstrating the controller’s ability to handle lateral disturbances and sensor noise.

### 4.6. System-Level Performance and Validation

The combined (WMR + RM + MVSS) results demonstrate that DT-TTSMC controller provides robust and precise trajectory tracking in both axes, and MCPRS operates reliably under hybrid task execution involving motion, manipulation, and synchronization. The integration of IKC-based motion planning, MVSS-based visual servoing, and real-time SCADA coordination, enables precise manipulation of components, reliable execution of pick-and-place operations, and seamless interaction between MCPRS and A/D/R MPL. Furthermore, the experimental results validate consistency between planned and executed trajectories, effectiveness of visual feedback in reducing positioning errors, and capability of the system to operate in real-time industrial scenarios. This approach ensures precision and high accuracy by DT-TTSMC + IKC + MVSS, flexibility by modular distributed control, scalability by OPC UA + IoT integration, intelligence by AI-based perception, decision support, educational value by real-time remote experimentation platform, scalability toward Industry 4.0/5.0, and IoT-enabled environments. Furthermore, the satisfaction of the H∞ performance bounds confirms that the closed-loop system maintains robustness under bounded disturbances, making it suitable for real-time industrial deployment within Industry 4.0/5.0 frameworks.

## 5. AI Optimization Layer for IoT–Cloud-Based Control of MPL Assisted by Dual CPRS

The AI optimization layer represents the intelligent decision-making component of the proposed cyber–physical architecture, ensuring adaptive, autonomous, and robust operation of the MPL assisted by the dual CPRS, composed of A/D/Replacement CPRS and the MCPRS. This layer is deployed above the real-time control and discrete-event coordination levels and is tightly integrated with the IoT–Cloud platform, DT environment, and SCADA/Node-RED supervision architecture. The main objective of the AI layer is to optimize production flow, reduce cycle time, improve task allocation, enhance fault tolerance, and support predictive and adaptive decision-making under uncertain manufacturing conditions.

### 5.1. Reinforcement Learning (RL) for Intelligent Scheduling and Autonomous Decision-Making

Reinforcement learning is introduced to enable autonomous optimization of production scheduling and dynamic task allocation between: CPRS, MCPRS (PeopleBot WMR, Cyton RM, MVSS), A/D ML, WS1–WS7, FC1, FC2. The RL agent continuously learns optimal actions based on system states acquired from PLC sensors, OPC-UA variables, Node-RED event states, SHPN markings, DT simulation results and camera-based visual feedback (MVSS). This allows the system to adapt online to transport delays, robot congestion, WS failures, storage conflicts, component unavailability and maintenance requirements, without requiring manual rescheduling.

1. The state vector consists of

(57) $$S=\left[M_{SHPN},X_{WMR},q_{RM},WH_{i},FC_{j},Sensor_{k},Fault_{l}\right]$$

where $M_{SHPN}$ is Petri Net marking, $X_{WMR}$ is mobile robot position, $q_{RM}$ is robot manipulator joint states. $WH_{i}$ is warehouse occupancy, $FC_{j}\;is$ flexible cell availability $Sensor_{k}$ is process sensor signals and $Fault_{l}$ is detected disturbances/failures. RL possible actions include start assembly of WP1/WP2, transport WPX to A/D ML, initiate disassembly, launch repair cycle, select replacement component, assign MCPRS trajectory, select warehouse destination, and schedule the ABB/Cyton manipulation task.

2. The RL reward function minimizes:

(58) $$J=\alpha T_{c}+\beta E_{c}+\gamma F_{r}+\delta D_{p}$$

where $T_{c}$ is cycle time, $E_{c}$ is energy consumption, $F_{r}$ is fault risk, and $D_{p}$ is delivery penalty, while maximizing productivity, flexibility, system availability, remanufacturing efficiency. RL provides adaptive scheduling, self-learning optimization, predictive maintenance support, autonomous decision-making, reduced operator intervention, and cloud-assisted optimization which are essential for Industry 5.0 intelligent manufacturing.

### 5.2. Type-3 Fuzzy Logic for IoT–Cloud-Based Control of MPL Assisted by Dual CPRS

To further improve robustness, uncertainty handling, human-centred supervision, and safe remote operation of the IoT–Cloud-based control of MPL assisted by Dual CPRS, a Type-3 Fuzzy Logic Extension (T3-FLS) is introduced as an intelligent supervisory layer above the real-time control structure. CPRS uncertainties are not only present in process variables but also in sensor confidence, communication reliability, camera visibility, operator decisions, cloud latency, cybersecurity trust levels, and maintenance prediction reliability. The Type-3 fuzzy supervisor is placed above continuous control layer (DT-TTSMC for PeopleBot WMR, IKC for Cyton RM, IBVS for MVSS), above the discrete-event layer, SHPN task synchronization, Node-RED task planning and OPC-UA scheduling logic, and below Cloud/AI optimization layer, RL optimization, predictive maintenance, DT simulation. This creates Cloud AI→Type-3 Fuzzy Supervisor→SHPN+Controllers→Real Process. Type-3 membership structure consists of μ (x, u, v), where x is the process, u is secondary uncertainty, and v is tertiary uncertainty. Example Rule Base:

- Rule 1: IF trajectory error is HIGH, network latency is HIGH obstacle proximity is dangerous THEN reduce WMR velocity significantly increase TTSMC gain, activate local autonomous mode.
- Rule 2: IF visual confidence is LOW, operator confidence is LOW, cyber trust score is low THEN disable remote manipulation, switch to safe local mode require manual confirmation.
- Rule 3: IF warehouse congestion is HIGH, and SHPN deadlock probability is HIGH THEN activate rescheduling trigger RL optimization.

1. Integration with DT-TTSMC. The sliding surface:

(59) $$s=\dot{e}+\lambda e$$

is modified using fuzzy adaptive gains:

(60) $$s=\dot{e}+\lambda_{f}e$$

where

(61) $$\lambda_{f}=T3FLS(e,\dot{e},Delay,Trust)$$

This produces reduced chattering, improved disturbance rejection, adaptive finite-time convergence.

2. Integration with IBVS. The image-based control law

(62) $$v=-\lambda L_{s}^{+}e$$

becomes

(63) $$v=-\lambda_{f}L_{s}^{+}e$$

where

(64) $$\lambda_{f}=T3FLS(ImageError,Visibility,Latency)$$

This improves visual stability, camera robustness and precision under poor visibility. T3-FLS enables safe IoT remote laboratories by local safe fallback. When cloud delay becomes critical $Delay>Delay_{critical}$, system switches to local autonomous control mode without operator intervention. If intrusion risk is detected, the $Trust<Trust_{threshold}$ system automatically isolates remote commands, blocks cloud actuation, and preserves local safe operation. In Education 5.0 mode, students can operate remotely, but T3-FLS guarantees forbidden commands rejection, collision prevention, and workspace protection. The integration of type-3 Fuzzy Logic with DT-TTSMC, IKC, IBVS, SHPN, DT, and Cloud AI creates a next-generation intelligent manufacturing architecture capable of autonomous adaptation, resilient remanufacturing, human-centred supervision, secure remote laboratories, and sustainable circular production which strongly advances the transition toward Industry 5.0, Education 5.0, and AI-driven smart factories.

### 5.3. Fractional-Order Control for Enhanced Dynamic Performance of PeopleBot WMR, MVSS, Cyton 1500 RM, and A/D/R MPL

To improve robustness, transient response, disturbance rejection, and precision in the IoT–Cloud-based control of MPL assisted by the Dual CPRS, a Fractional-Order Control (FOC) framework is introduced. The proposed fractional-order strategy is applied to:

- PeopleBot WMR for transportation tasks;
- Cyton 1500 RM for pick-and-place and replacement operations;
- MVSS with Logitech HD camera for fine positioning;
- A/D/R MPL discrete–continuous coordination layer.

This creates a high-performance hybrid control architecture combining $\mathrm{Fractional}\;\mathrm{Control},\mathrm{DT}\text{-}\mathrm{TTSMC},\mathrm{IKC},\mathrm{IBVS},H_{\infty },and\;\mathrm{SHPN}$, for Industry 4.0/5.0 intelligent manufacturing.

The fractional derivative is defined as $D^{\alpha }x\left(t\right)$ where 0<α<1 and α is the fractional order. Non-integer differentiation introduces memory behaviour.

1. Fractional DT-TTSMC for PeopleBot WMR based on WMR kinematic model.

- The WMR dynamics consists of

(65) $$\left\{\begin{array}{c} \dot{x}=\mathrm{vcos}\theta \\ y^{˙}=vsin\theta \\ \theta^{˙}=\omega \end{array}\right.,$$

where v: is linear velocity, and ω is angular velocity

- Tracking Error is defined as

(66) $$\left\{\begin{array}{c} e_{x}=x-x_{d}\\ e_{y}=y-y_{d}\\ e_{\theta }=\theta -\theta_{d} \end{array}\right..$$

- Fractional Sliding Surface. The classical surface

(67) $$s=\dot{e}+\lambda e$$

is extended to

(68) $$s=D^{\alpha }e+\lambda e,$$

where 0<α<1. This provides smoother convergence, better robustness, and reduced chattering:

- Fractional TTSMC law. The control law becomes

(69) $$u=-k_{1}|s{|}^{1/2}\mathrm{sign}(s)-k_{2}\int \mathrm{sign}(s)dt,$$

with $s=D^{\alpha }e+\lambda e.$ This ensures finite-time convergence, strong disturbance rejection, smooth tracking for MCPRS navigation.

2. Fractional IKC for Cyton 1500 RM. The differential motion is

(70) $${\dot{x}}_{e}=J(q)\dot{q},$$

where $x_{e}$ is the end-effector position, J(q) is Jacobian, and q is joint coordinates. Classical IKC $\dot{q}=J^{+}{\dot{x}}_{d}$ becomes fractional one

(71) $$D^{\beta }q=J^{+}{\dot{x}}_{d},$$

where 0<β<1. This improves joint smoothness, vibration suppression, and flexible link compensation, which is especially important for Cyton 1500.

3. Fractional IBVS for MVSS. The standard law for IBSV is $v=-\lambda L_{s}^{+}e,$ where $L_{s}$ is interaction matrix e is image feature error. Fractional visual servoing becomes

(72) $$v=-K_{p}e-K_{i}D^{-\mu }e,$$

where 0<μ<1. This fractional integral improves, robustness to lighting changes, smoother camera response, higher positioning precision, and better visual convergence for Logitech HD camera guidance.

4. Fractional H_∞_ Robustness. The system,

(73) $$D^{\alpha }x=Ax+Bu+Ew,$$

with disturbance w must satisfy

(74) $${∥z∥}_{2}<\gamma {∥w∥}_{2}.$$

This is solved via Fractional H_∞_ LMI for robust performance guarantees. This strongly improves cloud-delay robustness, sensor degradation tolerance and cybersecurity resilience.

5. Fractional SHPN coupling. The continuous places in SHPN are extended using fractional dynamics:

(75) $$D^{\alpha }P_{c}(t)=f(P_{c},T).$$

This models realistic transport delay, warehouse flow inertia, and continuous MCPRS movement more accurately than classical PN. Typical values for parameter tunning are α=0.7, β=0.8, μ=0.6 chosen using DT simulation, MATLAB optimization, RL-assisted tuning, and Node-RED adaptive scheduling.

**Figure 31:**
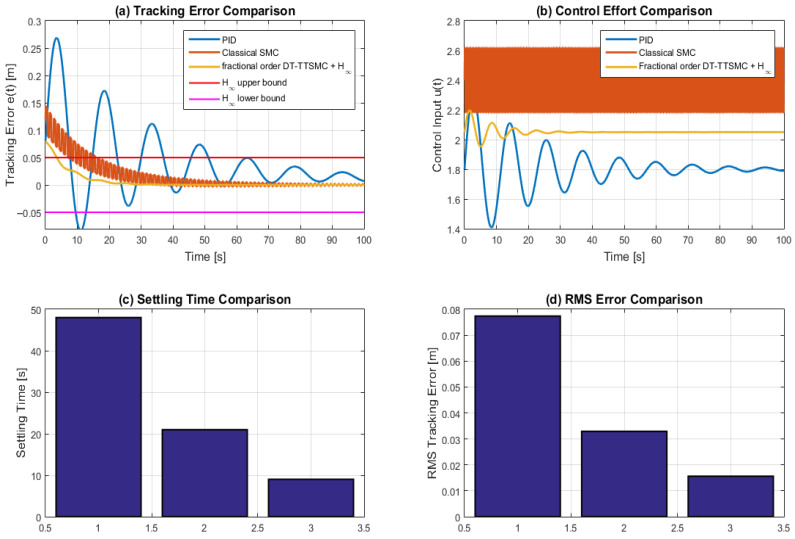
Three control strategies applied to the MCPRS during the component recovery: (**a**) tracking error evolution $e\left(t\right)$ for the three control approaches together with the upper and lower $H_{\infty }$ performance bounds, (**b**) the control effort $u\left(t\right)$ generated by the three controllers, (**c**) the settling time comparison, (**d**) RMS tracking error obtained for each controller.

Figure 31 presents a comparative performance evaluation of three control strategies applied to the MCPRS during the component recovery operation: conventional PID control, classical Sliding Mode Control (SMC), and the proposed fractional-order discrete-time terminal twisting sliding mode controller (fractional-order DT-TTSMC) combined with $H_{\infty }$ robustness. The comparison is carried out in terms of trajectory tracking accuracy, control effort, settling time, and RMS tracking error during the recovery mission executed by the PeopleBot WMR assisted by the MVSS and Cyton 1500 robotic manipulator. The proposed fractional-order DT-TTSMC + $H_{\infty }$ controller achieves the fastest convergence and the smoothest transient response. The tracking error rapidly converges toward zero while remaining fully confined within the prescribed $H_{\infty }$ robustness bounds, demonstrating superior robustness, finite-time convergence properties, and enhanced disturbance attenuation capability. The fractional-order dynamics additionally provide memory effects and smoother corrective actions, reducing oscillations and improving trajectory precision during the recovery task. The fractional-order DT-TTSMC + $H_{\infty }$ controller generates significantly smoother control inputs while preserving fast convergence and robustness.

The reduction in chattering and smoother actuator commands are particularly important for MCPRS operating under network delays, cloud communication uncertainties, and real-time industrial constraints.

The fractional-order DT-TTSMC + $H_{\infty }$ strategy achieves the shortest settling time, approximately 9 s, confirming the effectiveness of the terminal sliding mode formulation combined with fractional-order dynamics for rapid finite-time stabilization of the MCPRS during component recovery. Also, this controller achieves the minimum Root Mean Square (RMS) tracking error, demonstrating the highest trajectory tracking accuracy and robustness among all evaluated approaches.

Finally, the proposed fractional-order framework provides faster convergence than PID and classical SMC, lower chattering than standard Sliding Mode Control, better disturbance rejection under floor friction and payload changes, improved visual accuracy for pick-and-place operations, better manipulator smoothness for Cyton RM, and improved cloud robustness for remote IoT control.

## 6. Discussion

### 6.1. Circular Manufacturing and Sustainable Production

The proposed A/D/R MPL assisted by dual CPRS is designed to support the principles of circular manufacturing and sustainable production. Unlike traditional linear production systems, where defective products are discarded, the proposed architecture integrates disassembly, repair, and component recovery processes directly into the production workflow. In this framework, products that fail the quality test are not treated as waste but are redirected toward disassembly or repair operations. Components recovered during disassembly are transported and stored in dedicated warehouses, where they can be reused in future assembly processes. This approach contributes to material reuse, waste reduction, and improved resource efficiency, which are key objectives of sustainable manufacturing systems.

The integration of assembly, disassembly, and repair functionalities within the same production environment allows the MPL to operate as a closed-loop manufacturing system, in which product lifecycle management is extended beyond the initial assembly phase. As a result, the system supports remanufacturing strategies, enabling defective products to be repaired or reconfigured instead of being discarded.

### 6.2. Remanufacturing and Component Recovery

The inclusion of disassembly and repair operations transforms the proposed MPL into a platform capable of supporting remanufacturing processes. When a workpiece fails the quality test, it is stored in the designated warehouse and subsequently processed through disassembly or repair workflows.

During disassembly, the MCPRS retrieves reusable components and transports them to the appropriate storage locations within the A/D ML warehouses. These recovered components can later be reintegrated into new assembly operations. In cases where the product contains defective or incompatible components, the repair process replaces the faulty elements with appropriate parts before the product is returned to the production flow.

This capability introduces an important level of flexibility and sustainability to the production system. By enabling component recovery and selective replacement, the system minimizes material losses while maintaining product quality and functionality.

Furthermore, the use of the MCPRS allows the recovery process to be executed autonomously and efficiently, reducing manual intervention and enabling dynamic reconfiguration of production tasks.

### 6.3. AI-Based Optimization of the A/D/R System

The complexity of the integrated assembly–disassembly–repair workflow, combined with the coordination between the MPL and the MCPRS, creates opportunities for the application of AI techniques to optimize system performance.

One promising approach involves the use of reinforcement learning (RL) algorithms for adaptive task scheduling and dynamic resource allocation. In such a framework, the production system can learn optimal policies for assigning tasks to the MPL workstations and coordinating the MCPRS movements to minimize production time and resource consumption.

Another direction involves the integration of fuzzy logic-based decision systems, which are particularly suitable for handling uncertainties and incomplete information within complex manufacturing environments. Fuzzy decision mechanisms can assist in determining whether a workpiece should be directed toward repair, disassembly, or direct reuse, based on quality inspection results and system conditions.

In addition, fractional-order learning and control methods can be applied to improve the dynamic performance of robotic manipulation and mobile navigation within the MCPRS. Fractional-order controllers and learning strategies provide enhanced flexibility in modelling nonlinear system dynamics and can contribute to improved robustness in visual servoing and motion control tasks.

By integrating these AI-based optimization techniques with the SHPN-based Digital Twin framework, the system can evolve toward an intelligent adaptive manufacturing platform capable of self-optimization and predictive decision-making.

### 6.4. Integration with Education 4.0 and Education 5.0

The proposed A/D/R MPL assisted by dual CPRSs is implemented as a laboratory platform, designed not only for research in intelligent manufacturing systems but also for supporting advanced engineering education. The system provides an experimental environment that integrates robotics, automation, cyber–physical systems, DTs, and AI technologies within a unified framework.

From the perspective of Education 4.0, the laboratory system enables students and researchers to interact with modern industrial technologies that characterize Industry 4.0 environments. The platform combines several key technological elements typically encountered in smart manufacturing systems, including:

- Cyber–physical production systems (CPPS) through the integration of MPL and MCPRS,
- DT technology, linking the physical system with the virtual world,
- Industrial communication networks and PLC-based control,
- SCADA and HMI supervision systems,
- AR task planning using Node-RED dashboards,
- VR simulation using SHPN models,
- Mobile robotics and visual servoing systems.

By interacting with these technologies, students gain hands-on experience in designing and analyzing complex automation systems. The laboratory platform allows them to explore the complete lifecycle of intelligent manufacturing systems, from system modelling and simulation to implementation and real-time control. In addition, the integration of SHPN simulation and DT modelling enables students to test different production strategies and control algorithms in a virtual environment before implementing them on the physical system. This approach promotes experiential and problem-based learning, which is a fundamental principle of Education 4.0.

Beyond the technological focus of Education 4.0, the system also contributes to the emerging concept of Education 5.0, which emphasizes human-centred innovation, sustainability, and interdisciplinary collaboration. The A/D/R MPL architecture incorporates assembly, disassembly, and repair processes, enabling the study of circular manufacturing strategies and sustainable production practices. Through the integration of component recovery and remanufacturing operations, students can analyze how modern production systems can reduce waste and improve resource utilization.

Furthermore, the collaborative interaction between the MPL and the MCPRS demonstrates how human-supervised intelligent systems can operate within flexible and adaptive manufacturing environments. Students can study how autonomous robots, mobile platforms, and decision-support systems cooperate with human operators in the management of complex industrial processes.

The platform also supports interdisciplinary learning by combining knowledge from several domains, including:

- robotics and automation engineering;
- control systems and AI;
- industrial informatics and communication networks;
- manufacturing systems and sustainable production.

This interdisciplinary integration reflects the educational paradigm of Education 5.0, which aims to develop engineers capable of designing technologically advanced, sustainable, and socially responsible manufacturing systems.

### 6.5. Educational Benefits of the Digital Twin Laboratory

The DT architecture implemented for the A/D/R MPL assisted by dual CPRS provides significant pedagogical advantages. By combining the physical production system, the cyber control infrastructure, and the virtual simulation environment, the laboratory enables students to explore complex system behaviours from multiple perspectives.

The AR and VR tools allow learners to visualize production workflows, analyzed system dynamics, and understand the synchronization between discrete manufacturing processes and continuous robotic movements. At the same time, the SHPN modelling framework provides a formal methodology for representing hybrid systems and studying their dynamic behaviour.

Consequently, the laboratory platform serves as a comprehensive educational testbed for intelligent manufacturing systems, supporting both teaching and research activities in robotics, automation, and smart manufacturing.

### 6.6. Convergence of Industry 4.0/5.0 and Education 4.0/5.0

The proposed A/D/R MPL assisted by dual CPRS laboratory platform represents a convergence point between the paradigms of Industry 4.0, Industry 5.0, Education 4.0, and Education 5.0. From the Industry 4.0 perspective, the system integrates cyber–physical production systems, DT technology, intelligent robotics, and data-driven decision support to enable smart and flexible manufacturing processes. At the same time, the incorporation of assembly, disassembly, and repair functionalities, together with component recovery and remanufacturing capabilities, aligns the system with the principles of Industry 5.0, which emphasize sustainability, resilience, robustness, and human-centred production.

In parallel, the laboratory platform supports the objectives of Education 4.0 by providing students and researchers with direct access to advanced industrial technologies such as AR-based task planning, VR-based system simulation, hybrid Petri Net modelling, and mobile robotic manipulation. Furthermore, by incorporating sustainable manufacturing strategies and interdisciplinary collaboration between robotics, automation, artificial intelligence, and manufacturing engineering, the platform contributes to the broader vision of Education 5.0, which aims to develop engineers capable of designing intelligent, sustainable, and socially responsible industrial systems.

Consequently, the proposed architecture not only serves as a research framework for the development of intelligent manufacturing systems but also functions as an integrated educational testbed, supporting the training of future engineers in the design, modelling, and control of next-generation cyber–physical production systems.

### 6.7. Cybersecurity in IoT–Cloud-Based Control of A/D/R MPL Assisted by Dual CPRS

The proposed IoT–Cloud-based control architecture for the A/D/R MPL introduces multiple cyber–physical attack surfaces due to the integration of:

- Cloud/VPN remote access,
- OPC-UA industrial communication,
- Node-RED edge orchestration,
- DT synchronization (MATLAB/SHPN),
- AI-based decision-making modules,
- These interconnected layers expose the system to cyber–physical threats, including,
- Unauthorized access to PLCs (command injection),
- Data integrity attacks on sensor signals,
- Denial-of-service (DoS) on communication networks,
- Man-in-the-Middle (MitM) attacks on OPC-UA channels,
- Adversarial manipulation of AI-based scheduling,
- DT desynchronization attacks.

Given that the system directly controls robotic manipulation and mobile robotics (MCPRS), cybersecurity becomes safety critical.

A defense-in-depth strategy is adopted, structured across five layers:

1. Device & Field Layer (sensors, actuators, PLCs) consist of secure PLC programming (Siemens S7-1200, S7-300 protection levels), firmware integrity verification, and physical access control to A/D ML and CPRS stations.
2. Communication layer (OPC-UA, Industrial Ethernet). Use of OPC UA with end-to-end encryption, certificate-based authentication, secure session tokens, and network segmentation by virtual LAN separation (CPRS/MCPRS/SCADA/Cloud).
3. Edge layer (Node-RED gateway) by HTTPS + token authentication, disabled unused ports and nodes, input validation for all sensor data and sandboxing of JavaScript functions.
4. Cloud & IoT layer by secure VPN tunnelling (IPSec/OpenVPN), API authentication (OAuth 2.0/JWT tokens), data encryption at rest and in transit.
5. Application layer (DT&AI). Integrity validation between real system and DT, detection of abnormal deviations in SHPN state evolution, and secure AI pipelines by model validation and protection against adversarial inputs.

### 6.8. Final Perspective

The integration of DT technology, AR-based task planning, VR-based SHPN simulation, and MCPRS-assisted manipulation provides a comprehensive framework for the implementation of intelligent manufacturing systems. The proposed A/D/R MPL architecture supports flexible production, remanufacturing, and component recovery, enabling a transition from traditional linear production models to sustainable circular manufacturing systems.

Moreover, the incorporation of AI-based optimization techniques, such as reinforcement learning, fuzzy decision systems, and fractional-order learning, opens new possibilities for improving system autonomy, adaptability, and operational efficiency. These capabilities position the proposed architecture as a promising solution for next-generation manufacturing systems aligned with the principles of Industry 4.0 and Industry 5.0, where cyber–physical integration, intelligent decision-making, and sustainable resource utilization play a central role.

## 7. Conclusions

### 7.1. Summary of the Main Conclusions

This paper presents the design and analysis of a multifunctional A/D/R MPL assisted by a dual CPRS. The proposed system integrates assembly, disassembly, and repair operations within a unified production architecture, enabling flexible manufacturing and component recovery in a laboratory-scale industrial environment.

A DT framework was developed to represent the interaction between the physical production system, the cyber control infrastructure, and the virtual world used for monitoring, planning, and simulation.

The developed models support the simulation and validation of assembly, disassembly, and repair workflows, enabling the evaluation of system performance before the implementation of real-time control strategies.

From a manufacturing perspective, the proposed architecture supports the principles of circular and sustainable production, since defective products can be redirected toward disassembly or repair operations instead of being discarded. The recovery and reuse of components contribute to improved resource efficiency and reduced material waste.

Finally, the A/D/R MPL assisted by dual CPRS also serves as an advanced educational laboratory platform, supporting the objectives of Education 4.0 and Education 5.0. By integrating cyber–physical systems, DT technologies, hybrid modelling, robotics, and intelligent control, the platform provides a comprehensive environment for research and training in next-generation smart manufacturing systems.

### 7.2. Summary of the Main Contributions

The main contributions of this work can be summarized as follows:

- Design of a multifunctional A/D/R manufacturing architecture. A novel A/D/R MPL assisted by dual CPRS is proposed, enabling the integration of assembly, disassembly, and repair processes within a unified production environment.
- Integration of a DT framework for smart manufacturing. The proposed system integrates the physical layer, cyber layer, and virtual world, enabling real-time monitoring, task planning, and predictive simulation of the production system.
- AR-based task planning and supervision. An AR environment implemented with Node-RED is developed to support workflow visualization, operator interaction, and real-time supervision of MPL and MCPRS operations.
- Hybrid modelling of the integrated system using SHPN. The behaviour of the A/D/R MPL assisted by dual CPRS is modelled using SHPNs, combining STPNs for discrete manufacturing processes and CPNs for MCPRS displacement.
- Support for circular manufacturing and component recovery. The system architecture enables disassembly and repair workflows, allowing defective products to be redirected toward component recovery and reuse, thereby supporting sustainable and circular manufacturing strategies.
- Educational and research platform for Industry 4.0/5.0 technologies. The laboratory implementation of the A/D/R MPL assisted by dual CPRSs provides a comprehensive environment for research and education in cyber–physical production systems, Digital Twins, intelligent robotics, and sustainable robust manufacturing, aligning with the principles of Education 4.0 and Education 5.0.
- Real-time IoT–Cloud control of a dual CPRS architecture. A complete real-time control framework is developed for the MPL assisted by dual CPRS. The proposed system ensures seamless IoT–Cloud connectivity via OPC UA, distributed PLC, SCADA coordination, remote supervision and control in real time.
- Real-time integration of hybrid control (Continuous & Discrete). The work provides a fully implemented hybrid control architecture, combining continuous control TTSMC for WMR motion, inverse kinematics control (IKC) for manipulation, image-based visual servoing (IBVS) for precision positioning with discrete-event control, STPN for assembly, and SHPN for disassembly and repair.
- Real-time synchronization via PLCs signals and Petri Net events. The system introduces a formal synchronization mechanism between: PLC signals, Petri Net transitions, and robotic actions.
- AI optimization layer. The AI optimization layer represents the intelligent decision-making component of the proposed cyber–physical architecture, by RL, type-3 fuzzy logic, and FOC for ensuring adaptive, autonomous, and robust operation of the MPL assisted by the dual CPRS.
- Comparative performance evaluation of three control strategies applied to the MCPRS during the component recovery operation, conventional PID control, classical SMC, and the proposed FOC DT-TTSMC combined with $H_{\infty }$ robustness.
- A multilayer cybersecurity framework is integrated into the proposed IoT–Cloud-based control architecture to ensure secure and resilient operation of the A/D/R MPL assisted by dual CPRS. The approach combines secure OPC-UA communication, edge-level protection using Node-RED hardening, cloud security mechanisms, and DT validation through SHPN consistency checks. AI-based scheduling modules are safeguarded against adversarial manipulation, while intrusion detection mechanisms monitor system behaviour in real time. This comprehensive strategy ensures both cyber and physical safety, making the system suitable for Industry 4.0/5.0 and Education 4.0/5.0 environments.

### 7.3. Summary of the Main Future Research Directions

Future research will focus on:

- The integration of AI-based optimization methods, such as reinforcement learning, fuzzy decision systems, and fractional-order learning strategies, to improve task scheduling, adaptive control, and autonomous coordination between the MPL and the MCPRS.
- The development of adaptive and AI-assisted $H_{\infty }$ robust control strategies for the MCPRS and A/D/R MPL.
- The extension of IoT–Cloud-based A/D/R MPL toward fully autonomous predictive and self-healing manufacturing systems.
- The development of resilient and fault-tolerant Industry 5.0 architectures capable of operating under abnormal scenarios.
- The extension of the proposed DT architecture toward collaborative human-centred Industry/Education 5.0 environments.

## Data Availability

The original contributions presented in this study are included in the article. Further inquiries can be directed to the corresponding author.
